# Smad4-dependent morphogenic signals control the maturation and axonal targeting of basal vomeronasal sensory neurons to the accessory olfactory bulb

**DOI:** 10.1242/dev.184036

**Published:** 2020-04-27

**Authors:** Ankana S. Naik, Jennifer M. Lin, Ed Zandro M. Taroc, Raghu R. Katreddi, Jesus A. Frias, Alex A. Lemus, Morgan A. Sammons, Paolo E. Forni

**Affiliations:** Department of Biological Sciences; The RNA Institute; University at Albany, State University of New York, Albany, NY 12222, USA

**Keywords:** Axonal targeting, BMP, Glomeruli, Smad4, Olfactory, Vomeronasal sensory neurons

## Abstract

The vomeronasal organ (VNO) contains two main types of vomeronasal sensory neurons (VSNs) that express distinct vomeronasal receptor (VR) genes and localize to specific regions of the neuroepithelium. Morphogenic signals are crucial in defining neuronal identity and network formation; however, if and what signals control maturation and homeostasis of VSNs is largely unexplored. Here, we found transforming growth factor β (TGFβ) and bone morphogenetic protein (BMP) signal transduction in postnatal mice, with BMP signaling being restricted to basal VSNs and at the marginal zones of the VNO: the site of neurogenesis. Using different Smad4 conditional knockout mouse models, we disrupted canonical TGFβ/BMP signaling in either maturing basal VSNs (bVSNs) or all mature VSNs. Smad4 loss of function in immature bVSNs compromises dendritic knob formation, pheromone induced activation, correct glomeruli formation in the accessory olfactory bulb (AOB) and survival. However, Smad4 loss of function in all mature VSNs only compromises correct glomeruli formation in the posterior AOB. Our results indicate that Smad4-mediated signaling drives the functional maturation and connectivity of basal VSNs.

## Abstract (precis)

**Summary:** Genetic disruption of TGFβ/BMP signaling in maturing basal vomeronasal sensory neurons (VSNs) or in all mature VSNs indicates that Smad4 signaling drives maturation and connectivity of basal VSNs.

## INTRODUCTION

Neurons form complex and conserved neuronal circuits by innervating the dendritic compartments of specific target neurons. A current goal in neuroscience seeks to delineate the underlying mechanisms that establish and maintain neuronal identity, function and connectivity. The vomeronasal organ (VNO) is a specialized vertebrate olfactory subsystem used to detect pheromones (Cloutier et al., 2002; Dulac, 2000; Isogai et al., 2011; Mombaerts et al., 1996). Neurons of the vomeronasal epithelium (VNE) continue to form postnatally (de la Rosa-Prieto et al., 2010). As newly formed neurons mature, they innervate dendrites of specific second-order neurons in the accessory olfactory bulb (AOB) (Mombaerts et al., 1996). The sensory epithelium of the mouse VNO is composed of vomeronasal sensory neurons (VSNs) that selectively express receptors encoded by one of the two vomeronasal receptor (VR) gene families: V1r and V2r (Chamero et al., 2012). V2r-expressing neurons localize to the basal region of the VNE and target posterior regions of the AOB (pAOB), while V1r-expressing neurons reside in the apical region and project to anterior regions of the AOB (aAOB). These non-overlapping V1r- and V2r-expressing populations of VSNs bind different ligands, project to different areas of the AOB and trigger distinct innate behaviors.

Olfactory sensory neurons (OSNs) of the main olfactory epithelium (MOE) expressing a specific receptor converge upon two out of ∼1800 spatially invariant glomeruli within the olfactory bulb (Ressler et al., 1994; Vassar et al., 1994; Wang et al., 1998). This anatomical specificity differs in the vomeronasal system. In this olfactory subsystem, VSNs expressing the same receptor coalesce onto multiple glomeruli within spatially conserved regions of the AOB (Belluscio et al., 1999; Del Punta et al., 2002). To date, findings suggest that unidentified spatial cues in the nasal area influence OSN gene expression and target specificity (Coleman et al., 2019). However, our current knowledge of the underlying signals that establish the spatial (apical-basal) identity of VSNs and delineate specific synaptic partners remains limited.

Molecules belonging to the transforming growth factor (TGF) super-family, such as TGFβ, activin and bone morphogenic proteins (BMPs), control nervous system growth, differentiation, axonal growth patterning and neuronal network formation (Banerjee and Riordan, 2018; Le Dreau et al., 2012; Lee et al., 2000; Liem et al., 2000, 1995; Schmidt et al., 1995). In *Drosophila*, retrograde BMP signaling mediates neuromuscular junction synaptic growth and cytoarchitecture (Ball et al., 2010; Piccioli and Littleton, 2014). In rodents, BMP signaling also helps establish neuronal identity and define somatosensory map formation in the trigeminal nerve (Ball et al., 2010; Banerjee and Riordan, 2018; Berke et al., 2013; Fuentes-Medel and Budnik, 2010; Hegarty et al., 2013; Hodge et al., 2007; Liao et al., 2018; Piccioli and Littleton, 2014). Studies investigating specific roles for members of the TGFβ family in the formation of functional circuits of the VSNs with the brain have been quite limited.

Smad4 is a central signaling molecule in canonical TGFβ/BMP intracellular signaling. Upon ligand-receptor binding, Smad4 forms a complex with phosphorylated receptor regulated Smads (R-Smad), which then translocate to the nucleus to activate gene transcription. The R-Smads for the BMP pathway are Smad1, Smad5 and Smad8, whereas Smad2 and Smad3 are for the TGFβ pathway (Shi and Massagué, 2003). Activated Smads bind with other transcription factors to integrate signals from the TGFβ family with other signaling pathways (Hill, 2016). The binding affinity of BMP ligands for extracellular matrix components, such as collagen IV (Col-IV), define spatial BMP signaling gradients (Bunt et al., 2010; Garamszegi et al., 2010; Paralkar et al., 1991, 1992; Wang et al., 2008). In this study, we have analyzed TGFβ/BMP signaling in the VNO and found that BMP signal transduction is limited to the basal territories of the VNO and in the marginal zones, where VSNs are generated. To test the role of TGFβ/BMP signaling at different cellular contexts, we exploited AP-2εCre and OMPCre mouse lines as genetic entry points to disrupt Smad4 function in immature basal VSNs and in mature apical and basal VSNs, respectively. Our results suggest that Smad4-dependent morphogenic signals in immature basal VSNs are required to complete the cellular maturation of basal VSNs and that Smad4 dependent signaling in both immature and mature basal VSNs is crucial for proper circuit formation between the basal VSNs and the pAOB.

## RESULTS

### Localization of active BMP signaling in basal VSNs

**Fig. 1.**
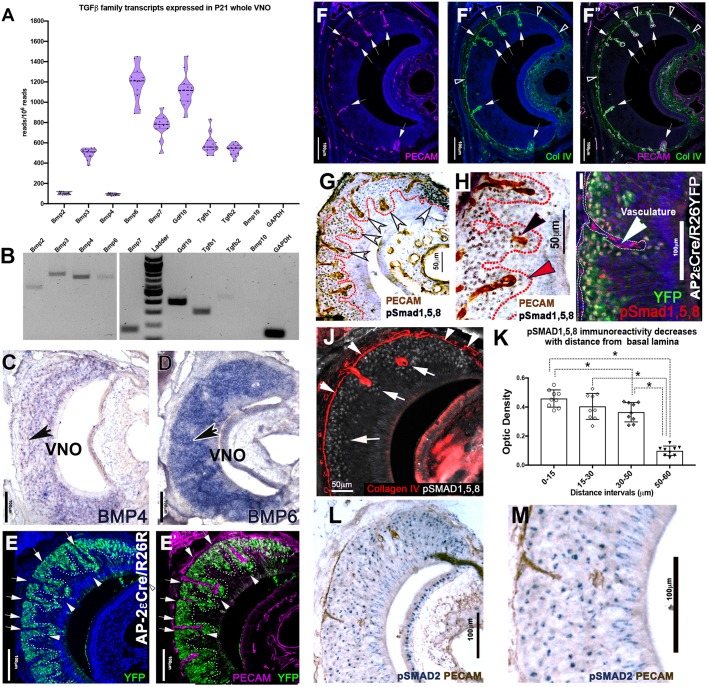
**TGF-β/BMP signaling in the VNO.** (A) Transcript abundance of BMP and TGFβ molecules according to RNASeq analysis. (B) RT-PCR confirmation of BMP and TGFβ molecules, the white gap distinguishes between two different parts of the same gel. (C,D) *In situ* hybridization for BMP4 (C) and BMP6 (D) on P15 VNO. Arrows indicate VSNs positive for BMP4 (C) and BMP6 (D) transcript. (E,E′) Immunofluorescence against AP-2εR26YFP (P15) lineage tracing, the vasculature marker PECAM (magenta) and DAPI (blue) shows a close spatial association of YFP^+^ (green) basal VSNs to vasculature (white arrowheads). Arrows indicate the vasculature. (F-F″) Immunofluorescence in wild type (P21) for PECAM (magenta) and collagen IV (ColIV, green) with DAPI (blue) shows the basal lamina positive for Col IV (black arrowheads) encapsulating the invading vasculature (white arrows). (G) Immunohistochemistry in wild type (P15) for p-Smad1,5,8 immunoreactivity (gray cells, white arrowheads) in VSNs proximal to the PECAM-positive (brown) vasculature-transducing BMP. (H) Magnification of G. Black arrowhead indicates PECAM^+^ vasculature; red arrowhead indicates p-Smad1,5,8-positive VSNs transducing BMP. (I) p-Smad1,5,8 (red) and AP-2εCre/R26RYFP lineage tracing (green). Basal VSNs, which are positive for AP-2εCre recombination (YFP), have strong BMP signal transduction. White arrowhead indicates vasculature positive for p-Smad1,5,8. (J) Collagen IV immunostaining (red) highlights the basement membrane (white arrows) and p-Smad1,5,8 cells (gray) have stronger immunoreactivity proximal to the sources of collagen IV (white arrowheads). (K) p-Smad1,5,8 optical density (OD) after DAB staining at four distance intervals from the basement membrane in the VNO; unpaired *t*-test, *P<0.05; data are mean±s.e.m., *n*=3 animals; individual points represent the average of 30 cells counted per interval/sections; three sections/animal. (L) Immunostaining using anti-p-Smad2 (blue) shows TGFβ signaling in the VNO with uniform immunoreactivity in all VSNs. (M) higher magnification of L showing uniform expression of p-Smad2 in the VNE.

We analyzed mRNA-seq data from postnatal VNO (GSE134492) and found that the VNE expresses multiple molecules of the BMP/TGFβ family. RT-PCR using primers for select BMP/TGFβ family members produced amplicon products of the predicted size (Table S1), confirming the expression of the identified genes (Fig. 1A,B). We arbitrarily decided to document BMP4 and BMP6 RNA expression in tissue, as they possibly showed differential expression. *In situ* hybridization against BMP4 and BMP6 showed their expression levels in apical and basal territories of the VNE (Fig. 1C,D). Extracellular matrix components, such as collagen IV (Col IV), also participate in triggering active BMP signaling by sequestering or immobilizing morphogens and facilitating receptor binding (Bunt et al., 2010; Garamszegi et al., 2010; Paralkar et al., 1991, 1992; Wang et al., 2008). Col IV and PECAM immunostaining indicated collagen IV expression in the basement membrane of the VNO and around PECAM^+^ vasculature that invades the basal regions of the VNO (Fig. 1F-F″).

If BMP signaling differentially regulates survival, maturation or function of the two main types of VSNs, we predict the existence of differential signaling in these two population. We therefore examined BMP and TGFβ intracellular signaling using immunostaining against p-Smad1,5,8 and p-Smad2, respectively. Our immunostaining results showed immunoreactivity for p-Smad1,5,8 in PECAM-positive endothelial cells (Fig. 1I) and in VSNs in the basal territories proximal to PECAM^+^/Col IV^+^ vessels and basal lamina (Fig. 1G,H). We then confirmed that BMP signaling localized to only the basal VSNs using sections from AP-2εCre^+/−^/R26YFP lineage-traced animals (Lin et al., 2018), in which only basal cells undergo Cre recombination (Fig. 1I). Double immunofluorescent staining against p-Smad1,5,8 and YFP showed that nearly all basal VSNs were positive for active BMP signaling that began at the marginal zone (Fig. 1I, Fig. S1B,D). We detected only background levels of p-Smad1,5,8 immunoreactivity in the apical territories. In contrast, p-Smad2 immunostaining indicated active TGF-β signaling in both apical and basal territories (Fig. 1L,M). As the pattern of BMP signaling appeared stronger in the basal VSNs more proximal to the Col IV-rich basement membrane and vasculature (Fig. 1G,H), we performed a densitometric analysis of p-Smad1,5,8 cell immunoreactivity in relation to the distance from the basement membrane. We found a negative correlation between the expression of p-Smad1/5/8 and the distance from the basement membrane (Fig. 1J,K). These data suggest that only basal VSNs transduce BMP, with higher levels of pSmad 1,5,8 activation in the basal VSNs proximal to the basement membrane.

Retrograde BMP signaling in neurons can control synaptic growth at neuromuscular junctions and somatosensory map formation of the trigeminal nerve in rodents (Ball et al., 2010; Banerjee and Riordan, 2018; Berke et al., 2013; Fuentes-Medel and Budnik, 2010; Hegarty et al., 2013; Hodge et al., 2007; Liao et al., 2018; Piccioli and Littleton, 2014). Arx-1 null mice lack an olfactory bulb and virtually have no connection between VSNs and the brain (Taroc et al., 2017). We analyzed the pattern of p-Smad1,5,8 immunoreactivity in the VNO of these mice and we found a similar pattern of p-Smad1,5,8 immunoreactivity as in control animals (data not shown). These data suggest that the identified p-Smad 1,5,8 immunoreactivity in the VNO reflects locally produced morphogenic signals rather than retrograde signaling, as shown for other systems (Ji and Jaffrey, 2012).

### Maturing VSNs show active BMP signaling in marginal regions of the VNO

In addition to basal VSN-specific BMP signal transduction, our densitometric analysis also revealed significantly stronger p-Smad1,5,8 immunoreactivity in marginal regions of the VNO compared with medial regions (Fig. S1B). The marginal regions contain the immature basal VSNs positive for growth-associated protein 43 (GAP43) and AP-2ε but negative for olfactory marker protein (OMP) (Lin et al., 2018); immature apical VSNs positive for GAP43; and apical VSN specific transcription factor Meis2 positive and negative for OMP (Fig. S1A,C,D,F,G) (Brann and Firestein, 2010; Enomoto et al., 2011; Giacobini et al., 2000). We then quantified p-Smad1,5,8, AP-2ε and GAP43 immunoreactivity in marginal versus medial regions of the olfactory epithelium (Fig. S1B-D). We observed a higher level of expression for all three markers in marginal regions of the VNO compared with medial regions where VSNs reach maturity (OMP^+^) (Fig. S1B,C,F). In contrast, immunostaining and quantification for the active TGFβ signaling effector molecule p-Smad2 and the apical marker Meis2 did not show differences in marginal and medial regions of the VNE (Fig. S1E,G). The presence of TGFβ/BMP signal transduction in the VNE and the different levels of pSmad1,5,8 immunoreactivity in immature VSNs (marginal zones) and in mature basal VSNs (medial basal zone) prompted us to investigate whether BMP controls maturation or homeostasis of basal VSNs.

### Conditional ablation of Smad4 disrupts TGF-β/BMP signaling in basal VSNs

**Fig. 2.**
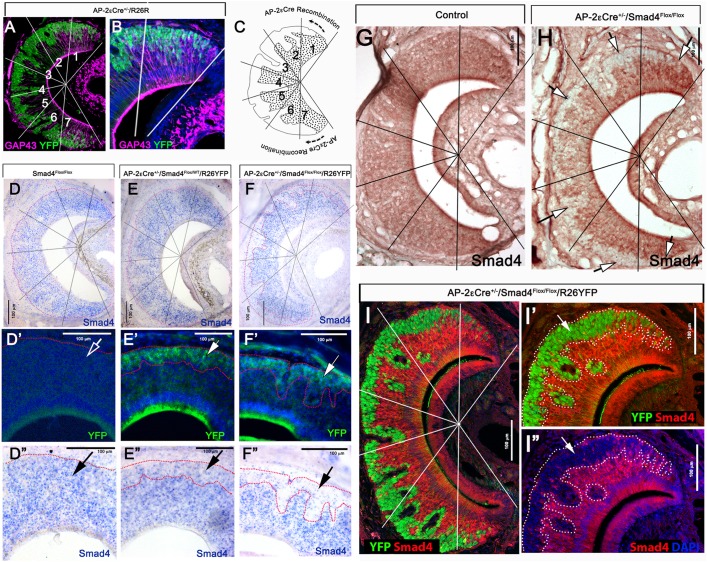
**Smad4 ablation in differentiated immature basal VSNs.** (A,B) Immunostaining of AP-2εCre^+/−^/R26YFP (P15) for the immature VSN marker GAP43 (magenta), YFP (green) and DAPI (blue), highlighting AP-2εCre recombination in basal VSNs. (B) Magnification of A. (C) Cartoon illustrating AP-2εCre recombination in basal VSNs. Lines and numbers indicate the seven different sectors of the VNO, where sectors 1 and 7 are the marginal regions, which is where neurogenesis occurs, and 2-6 are medial. (D-F) *In situ* hybridization for exon 8 of Smad4 in P15 control (D), P15 Smad4 heterozygous tracing control (E) and in P15 Smad4 homozygous traced cKO (F). (D′,E′,F′) AP-2εCre recombination marked by YFP immunostaining in P15 control (D′), P15 Smad4 heterozygous tracing control (E′) and in P15 Smad4 homozygous traced cKO (F′). (D″,E″,F″) YFP immunostaining highlighting AP-2εCre recombination and Smad4 exon 8 *in situ* hybridization showing uniform expression of a Smad4 exon 8 transcript in the VNE in P15 control (D″), lower expression of Smad4 transcript in lineage-traced basal VSNs in comparison with apical VSNs in P15 Smad4 heterozygous tracing control (E″), and almost no expression of Smad4 transcript in traced basal VSNs in P15 Smad4 traced cKO (F″). White arrows indicate Cre recombination (YFP). Black arrows indicate detectable or non detectable Smad4. (G,H) Immunostaining for Smad4 in control (G) and cKO (H). White arrows indicate complete lack of Smad4 in basal VSN. (I-I″) YFP (green) and Smad4 (red) immunostaining shows AP-2εCre recombination in P15 Smad4 homozygous traced cKO.

To elucidate the role of canonical TGF-β/BMP signaling in differentiating basal VSNs, we exploited AP-2εCre mice as a genetic entry point to conditionally ablate Smad4^flox/flox^ (Yang et al., 2002) in immature basal VSNs (Lin et al., 2018). We validated AP-2εCre mediated Smad4 ablation at postnatal age P15. The VNO at this stage is functional and close to its final size, but still retains a considerable amount of immature neurons generated during the postnatal proliferation peak (Wakabayashi and Ichikawa, 2007). To validate the ablation of functional Smad4, we performed *in situ* hybridization using an RNA probe against exon 8 of the Smad4 gene, which is flanked by LoxP sites (Yang et al., 2002), and immunohistochemistry against Smad4 (Benazet et al., 2012; Yang et al., 2002). Smad4 mRNA and protein expression analysis on Smad4^flox/flox^ controls, triple heterozygous mutants AP-2εCre^+/−^/Smad4^WT/flox^/R26^YFP+/−^ and traced conditional KOs AP-2εCre^+/−^/Smad4^flox/flox^/R26^YFP+/−^ verified Smad4 ablation in the cells that underwent Cre-mediated recombination (Fig. 2D-F″).

In Smad4^flox/flox^ control animals, visual inspection indicated comparable Smad4 mRNA levels in both basal and apical territories of the VNO (Fig. 2D-D″). In triple heterozygous AP-2εCre^+/−^/Smad4^flox/WT^/R26^YFP+/−^ mice, we detected weaker Smad4 expression in basal domains compared with apical regions, likely reflecting the loss of one allele (Fig. 2E-E″). In the conditional KO, most cells positive for AP-2εCre recombination in the basal domain marked by YFP were negative for Smad4 transcript expression (Fig. 2F-F″). Using immunolabeling against Smad4, we confirmed the loss of functional Smad4 protein expression in basal territories starting at the marginal regions (Fig. 2G,H). Using Smad4 and YFP double-immunolabeling on sections from AP-2εCre^+/−^/Smad4^flox/flox^/R26^YFP+/−^ mice, we quantified the recombination efficiency at ∼98%±0.716% (Fig. 2I-I″) starting at the marginal zones of the VNO, which contains immature neurons (use Fig. S1A,B as a reference). Our data confirmed the high specificity and penetrance of Cre-mediated Smad4 ablation in maturing basal VSNs.

### AP-2εCre driven Smad4 ablation does not alter the cellular composition of the VNO 2 weeks after birth

**Fig. 3.**
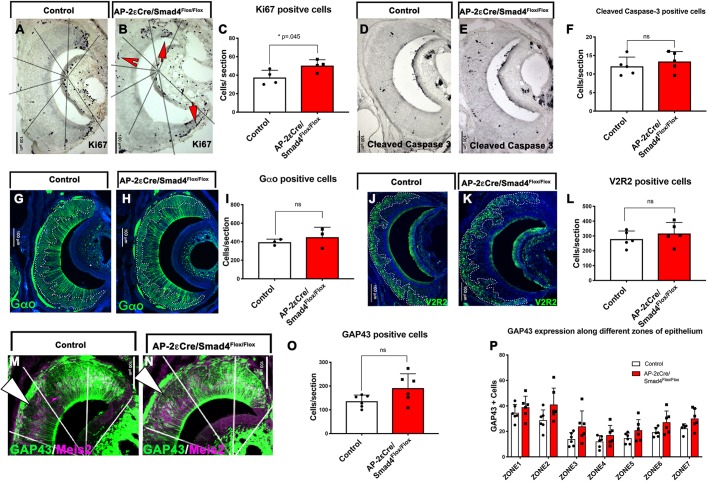
**Characterization of AP-2εCreSmad4 at P15 shows minute changes in the development of VSNs.** (A,B) Ki67 immunostaining in control (Smad4^flox/flox^) (A) and cKO (B). Red arrows and red arrowhead indicate proliferative cells in marginal and medial regions, respectively. (C) A significant increase in the number of proliferative cells was observed in the cKO (*n*=4). (D,E) Immunostaining for cleaved caspase 3 in control (D) and cKO (E). (F) No difference was observed in apoptosis between control and cKO (*n*=5). (G,H) Immunostaining for the basal marker Gαo in control (G) and cKO (H). (I) No significant difference was observed in the number of Gαo^+^ cells between genotypes (*n*=3). (J,K) Immunostaining for V2R2 in control (J) and cKO (K). (L) No difference in the number of V2R2^+^ cells was observed between genotypes (*n*=5). (M,N) Immunostaining using anti-GAP43 (green) and for the apical marker Meis2 (magenta) in control (M) and cKO (N). (O) A trend towards an overall increase was observed in the number of GAP43-positive immature neurons in cKO (*n*=6). (P) The number of GAP43-positive cells in marginal, intermediate and central zones among genotypes. Statistical analysis was carried out using a two-tailed unpaired *t*-test. Data are mean±s.e.m.

The first 2 postnatal weeks are a crucial period for development and growth of the VNO and the accessory olfactory bulb (Hovis et al., 2012). We analyzed the cell composition and molecular expression of VSNs at P15. At this stage, we found a small but significant increase in the number of proliferative (Ki67^+^) cells in the VNE of AP-2εCre^+/−^/Smad4^flox/flox^ (AP-2εCre/Smad4cKO). Even though these results could suggest a compensatory response to epithelial cell loss (Lin et al., 2018) (Fig. 3A-C), we did not detect significant changes in the number of cells positive for the apoptotic marker cleaved caspase 3 (Fig. 3D-F). Quantification of immunostaining against Gαo indicated that the localization and number of basal VSNs remained unchanged in AP-2εCre^+/−^/Smad4^flox/flox^ mice. We also found no changes in the expression of the basal receptor V2R2 (Fig. 3G-L). However, we did observe slightly more cells immunoreactive for GAP43 in the basal territories of the VNE of the AP-2εCre^+/−^/Smad4^flox/flox^ mice (Fig. 3M-P). These data indicate that Smad4 ablation in maturing basal VSNs, as assayed at P15, does not directly compromise the expression of basal-specific proteins, the neuronal position of VSNs in the epithelium or the neuronal cell number.

### Conditional Smad4 ablation in maturing basal VSNs produces a severe loss of basal neurons in adult mice

**Fig. 4.**
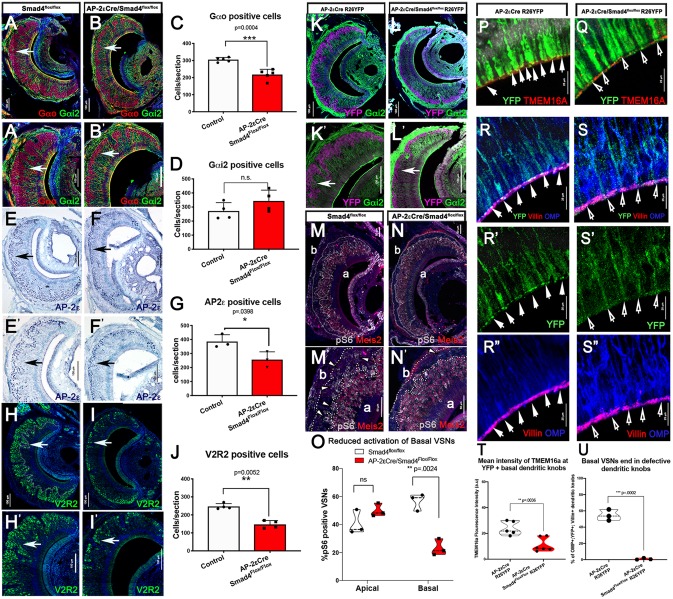
**The AP-2εCreSmad4cKO at P60 reveals a progressive loss of basal VSNs, reduced odor response and aberrant dendritic knob formation.** (A,B) Immunostaining for the basal marker Gαo (labeled in red, white arrow) and the apical marker Gαi2 (green) in control (Smad4^flox/flox^) (A) and cKO (B). (A′,B′) Magnification of A and B, respectively. (C) Graph representing a significant reduction in the number of Gαo-positive cells in the cKO (*n*=5). (D) Graph representing a non-significant increase in the number of Gαi2-positive cells (*n*=4). (E,F) Immunostaining for AP-2ε (black arrows) in control (E) and cKO (F). (E′,F′) Magnification of E and F, respectively. (G) Graph representing a significant reduction in the number of AP-2ε^+^ cells in the cKO (*n*=3). (H,I) Immunostaining for V2R2 (white arrows) in control (H) and cKO (I). (H′,I′) Magnification of H and I, respectively. (J) Reduction in the number of V2R2-positive cells in the cKO (*n*=4). (K,L) Immunostaining for AP-2εCre recombination (YFP, magenta) (white arrow) and the apical marker Gαi2 (green) in control (K) and cKO (L). (K′,L′) Magnification of K and L. (M-N′) Immunostaining for pS6 (gray) and the apical VSN marker Meis2 (red) in control (M; higher magnification in M′) and cKO (N; higher magnification in N′); a and b, apical and basal, respectively. Arrowheads indicate pS6^+^ basal VSNs. (O) Quantification of the percentage of pS6-positive apical and basal VSNs in control and cKO shows a significant reduction in the percentage of pS6-positive basal VSNs in the cKO (*n*=3). Violin plots indicating the mean percentage of pS6^+^ VSNs/biological replicate. (P,Q) Immunostaining for YFP and TMEM16A highlights the dendritic knobs in the apical regions of the VNE of controls (AP-2εCre^+/−^;R26YFP) (P, arrows). No or low TMEM16A expression was found on the few recognizable knobs of AP-2εCreSmad4CKO basal cells (Q, arrows). (R-S″) YFP, OMP and villin immunoreactivity in the dendritic knobs in controls (see arrows in R-R″). No colocalization (S) or well-defined knobs (compare R′ and S′) was identified in traced basal cells in AP-2εCreSmad4CKO (S′ and S″, arrows; R-S″). (T) Quantification of mean intensity of TMEM16a immunofluorescence in the knobs of controls and AP-2εCreSmad4CKO. Violin plots indicating mean TMEM16a fluorescence intensity/biological replicate. (U) Average fluorescence levels of basal knobs of controls were used as a parameter to identify ‘normal’ dendritic knobs (see Materials and Methods). Quantification of OMP; YFP^+^ basal knobs over the total number of villin^+^/OMP^+^ knobs/section showed that around 50% of the knobs in controls were basal. In AP-2εCre/Smad4 cKOs, few basal knobs were comparable with controls. Violin plots indicating the mean percentage of basal dendritic knobs/biological replicate. Statistical analysis was carried out using a two-tailed unpaired *t*-test.

Using our validated AP-2εCre/Smad4cKO, we examined whether Smad4 ablation in immature basal VSNs affects the homeostasis and/or connectivity of basal VSNs over time. This time dependency is crucial, as postnatal growth of the VNO and response to stimuli refines synaptic connections to the AOB (Hovis et al., 2012; Roos et al., 1988; Weiler et al., 1999). We therefore analyzed AP-2εCre^+/−^/Smad4^flox/flox^ and relative controls (Smad4^flox/flox^) at P60, which is the stage when most VSNs have formed functional connections (Hovis et al., 2012). At P60 we detected a 30-50% reduction in the number of VSNs expressing the basal VSN markers Gαo, V2R2 and AP-2ε; however, we did not detect changes in the number of Gαi2-positive apical VSNs (Fig. 4A-J).

We then sought to determine whether basal cells in AP-2εCre/Smad4cKO mice transdifferentiated into apical VSNs (Lin et al., 2018). AP-2εCre^+/−^/R26R lineage tracing on Smad4 conditional mutants (AP-2εCre^+/−^/Smad4^flox/flox^/R26^YFP+/−^) did not show the presence of traced VSNs that have transdifferentiated from basal to apical VSNs (Fig. 4K-L′). By analyzing GAP43 expression at P60 we observed increased immunoreactivity in the medial basal region of the VNO of the AP-2εCre^+/−^/Smad4^flox/flox^ (Fig. S2A,B,E,F,I,J). By performing Smad4 and GAP43 immunostaining on AP-2εCre^+/−^/R26RYFP control and AP-2εCre^+/−^/R26R^YFP+/−^/Smad4^flox/flox^ mice, we verified that elevated GAP43 immunoreactivity was limited to basal neurons that underwent AP-2εCre mediated recombination (Fig. S2C,D,G,H). Double OMP/GAP43 immunostaining showed GAP43 immunoreactivity in mature basal VSNs in AP-2εCre^+/−^/Smad4^flox/flox^ mice, while controls only showed sparse double-positive cells (Fig. S2K-N). Taken together, these data show that Smad4 ablation in maturing basal VSNs compromises maturation and long-term survival of basal VSNs.

### Smad4 ablation in maturing basal VSNs compromises neuronal response to odor stimuli

Impaired vomeronasal signal transduction leads to a slow and progressive cell loss during postnatal life. Consistent with observations in other mutants with reduced chemosensory detection (Chamero et al., 2011; Montani et al., 2013; Stowers et al., 2002), in AP-2εCre^+/−^/Smad4^flox/flox^ mice, we observed that basal VSNs form (Fig. 3) but decrease in number over time (Fig. 4C-G). As reported by others (Montani et al., 2013), we could not relate the slow reduction in cell number with an acute increase in apoptosis. To determine if the observed cell loss was related to impaired functionality of VSNs, we tested whether VSNs in mutant mice were able to detect pheromones. Pheromones carried by whole-male urine activate both basal and apical VSNs (Silvotti et al., 2018). Stimulation of VSNs by pheromones induces sustained phosphorylation of S6 ribosomal protein (pS6), which can serve as an indirect marker of neuronal activity (Silvotti et al., 2018). We assayed the functionality of VSNs by exposing male AP-2εCre^+/−^/Smad4^flox/flox^ and Smad4^flox/flox^ controls to whole-male urine and assayed pS6 levels 90 min after exposure. Quantification of pS6^+^ cells showed that, in the mutants, where we observed a 30-50% reduction in the number of basal VSNs (Fig. 4M-O), only ∼23% of remaining cells were able to respond to whole-male urine stimuli. These data indicate that the lack of Smad4 starting from immature stages compromised the ability of basal VSNs to detect and respond to pheromones (Fig. 4M-O).

### Basal neurons of AP-2εCre^+/−^/Smad4^flox/flox^ mutants have aberrant dendritic knobs

We then examined the mechanism underlying the impaired function of basal VSNs in AP-2εCre^+/−^/Smad4^flox/flox^ mice. Pheromone detection requires the interaction of molecules with the VRs along the apical microvilli. The VNO receptor potential in response to odorants is generated in the microvilli and dendritic knobs. The dendritic knobs of all VSNs are located at the apical surface of the VNE. By comparing transcriptome data for AP-2εCre^+/−^/Smad4^flox/flox^ with wild type and AP-2εCre^+/−^ controls, we found that AP-2εCre^+/−^/Smad4^flox/flox^ have a significant reduction in mRNA levels for the calcium-activated chloride channel (CaCC) TMEM16A/anoctamin 1, which is encoded by the gene *Ano1* (Francia et al., 2014; Münch et al., 2018). TMEM16A is expressed on the vomeronasal dendritic microvilli (Höfer et al., 2000; Münch et al., 2018). We performed immunostaining for TMEM16A on R26^YFP^ traced Smad4cKO (AP-2εCre^+/−^/R26R^YFP+/−^/Smad4^flox/flox^) and AP-2εCre controls (AP-2εCre^+/−^/R26R^YFP+/−^). This analysis confirmed a significant reduction in levels of TMEM16A immunoreactivity in the dendritic knobs of basal VSNs of AP-2εCre^+/−^/R26R^YFP+/−^/Smad4^flox/flox^ compared with controls (Fig. 4P,Q,T).

When performing this analysis, we realized that the dendritic knobs were harder to identify in the conditional KOs, whereas they appeared clear and distinct in controls (Fig. 4P-S′, arrows). To determine whether AP-2εCre^+/−^/Smad4^flox/flox^ had overall defective dendritic knobs, we performed immunostaining against OMP, the microvilli marker villin and YFP (Fig. 4R-S″). All knobs were identified via OMP;villin colocalization, while the knobs of the basal neurons were identified using OMP, villin and YFP colocalization. As expected, in control animals, the basal dendritic knobs constituted around 50% of the total knobs (Fig. 4U). Using the intensity of YFP immunoreactivity of controls as an arbitrary cutoff for normal knobs, we found that only a small percentage of the basal dendritic knobs of the cKO were comparable with the ones of controls (Fig. 4U).

Based on quantification for TMEM16A and villin at P60, our results suggest that loss of Smad4 activity in maturing VSNs compromises normal dendritic knob formation, which then impairs the functionality of basal VSNs. Notably similar defects in the basal dendritic knobs were observed at P15 (not shown), when no differences in cell numbers were found (Fig. 3). Overall, these data suggest that aberrant gene expression and defective neuronal development precedes and likely causes the reduction of basal VSNs over time.

### Smad4 ablation in basal VSNs compromises the formation of glomeruli in the pAOB

**Fig. 5.**
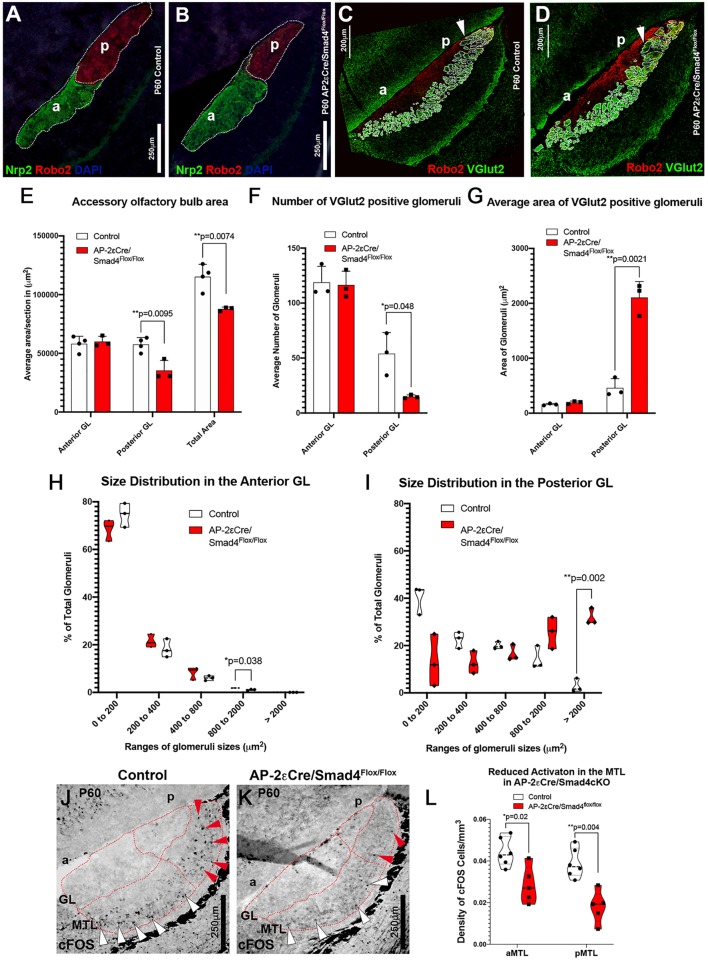
**pAOBs of AP-2εCre/Smad4cKO are smaller and have aberrant glomeruli.** (A,B) Immunofluorescence against Nrp2 and Robo2 on (A) P60 control (Smad4^flox/flox^) and (B) P60 AP-2εCre Smad4 cKO. a, anterior; p, posterior. (E) Quantification shows a reduced pAOB area and a reduction in the total area occupied by VSN fibers in the AOB of cKOs (control *n*=4, cKO *n*=3). Data are mean±s.e.m. (C,D) Immunostaining against Robo2 and VGlut2 highlights the glomeruli in the pAOB of controls (C, arrow) and cKOs (D, arrow). In the Smad4 cKOs, few and larger glomeruli were detected in the pAOB, whereas aAOB appeared to be unaffected. (F,G) Quantification of the average number and areas of the glomeruli in aAOB and pAOB of controls and AP-2εCre^+/−^/Smad4cKO (*n*=3). Data are mean±s.e.m. (H,I) The percentage of the number of glomeruli were binned in area ranges and quantified. The graphs (*n*=3) show an increase in the number of glomeruli with increased area in the pAOB (I) with a minor change in aAOB (H). Violin plot indicating the mean percentage of total glomeruli in size ranges/biological replicate. (J-L) Immunostaining (J,K) and quantification (L) for Fos on P60 control (Smad4^flox/flox^) and P60 AP-2εCre^+/−^/Smad4^flox/flox^, shows reduced activation in anterior (white arrowheads) and posterior (red arrowheads) MTL in AP-2εCre/Smad4cKO (control *n*=6, cKO *n*=5). Violin plot indicating the mean density of Fos cells/biological replicate. Statistical analysis was carried out using a two-tailed unpaired *t*-test.

GAP43 expression occurs in immature neurons that form an axon, undergo synaptic targeting or retain some plasticity (Enomoto et al., 2011; Strittmatter et al., 1990, 1992). Data from AP-2εCre^+/−^/Smad4^flox/flox^ mice indicated increased GAP43 immunoreactivity in basal VSNs (Fig. S2). To determine whether the lack of Smad4 signaling alters axonal formation or targeting, we analyzed VSN projections to the AOB. Immunostaining against the basal axonal marker Robo2 (Prince et al., 2009) and apical axonal marker Nrp2 indicated a ∼50% reduction in the area occupied by basal neuronal projections in the pAOB, consistent with reduced numbers of basal VSNs (Fig. 5E). No changes were observed in the aAOB (Fig. 5E). VSNs form synapses with dendrites of second-order neurons within organized glomeruli of the AOB. Basal VSNs form synapses in the posterior region of the AOB (pAOB), while apical neurons project to the aAOB (Brignall and Cloutier, 2015; Prince et al., 2013, 2009). We then determined whether Smad4 signaling is important for axonal organization by immunostaining against OMP, Robo2 and the presynaptic marker vesicular glutamate transporter 2 (VGlut2). Even though these results showed well-defined glomeruli in the anterior and pAOB of control animals, we detected fewer and larger glomeruli in the pAOB of AP-2εCre^+/−^/Smad4^flox/flox^ (Fig. 5C,D,F,G).

Neuronal loss in the basal VNO in adult mice and the abnormal number and size of the glomeruli suggest that Smad4 ablation in maturing VSNs compromises the long-term homeostasis of VSNs and their axonal convergence to specific glomeruli (Prince et al., 2013). To test whether basal VSNs and mitral cells in the AOB can form any functional connection, we analyzed the anterior and posterior mitral/tufted cell layer (MTL) of male animals after whole-male urine exposure. *Fos* is an immediate early gene broadly used as a marker for neuronal activation in AOB (Kumar et al., 1999). By performing Fos immunostaining after urine exposure, we found a dramatic reduction in its levels in activated mitral cells of the pAOB of the AP-2εCre^+/−^/Smad4^flox/flox^ mice; however, we also detected a reduction in activated mitral cells in the aAOB (Fig. 5J-L). These data suggest that Smad4 ablation in basal VSNs compromises VSNs homeostasis and pheromone-induced neuronal activation. However, Smad4 ablation does not completely prevent the formation of functional synapses, as some signs of synaptic activity were still found in the AOB. The reduction in Fos expression in activated mitral cells in the aAOB could indicate a postsynaptic defect secondary to Smad4 ablation in the mitral cells of the aAOB. In fact, we have previously reported that some mitral cells of the AOB are positive for AP-2ε lineage (Lin et al., 2018).

### Smad4 ablation in mature VSNs

**Fig. 6.**
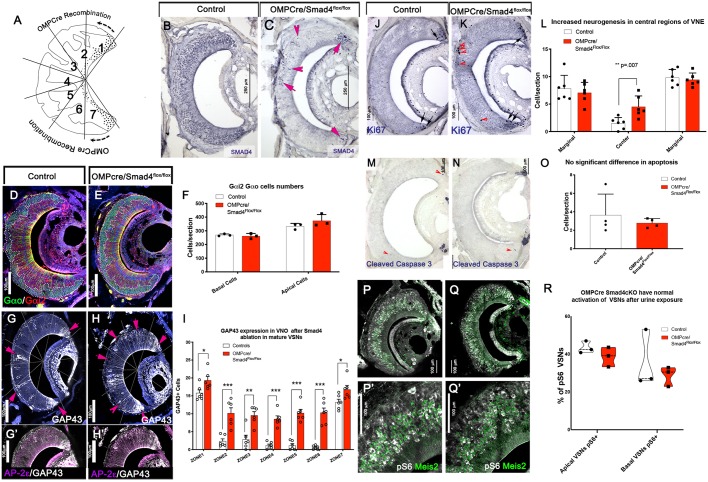
**Increased GAP43 immunoreactivity after Smad4 ablation in mature VSNs.** (A) Cartoon illustrating OMPCre recombination. (B,C) Immunohistochemistry against Smad4 in control (B) and cKO (C). Few Smad4-positive cells remain in the cKO (arrows), as they were OMP negative. (D-F) Immunostaining (D,E) and quantification (F) for the basal marker Gαo (green) and the apical marker Gαi2 (red). Quantification (F) shows no change in the number of Gαo and Gαi2 VSNs in cKO compared with controls (Smad4^flox/flox^) (*n*=3). Data are mean±s.e.m. (G,H) GAP43 immunostaining in control (G) and in cKO (H). Arrows indicate GAP43 immunoreactivity in VSNs from the medial zones (zones 2-6) of the VNE. (G′,H′) Immunostaining for AP-2ε and GAP43 indicate that GAP43-positive VSNs are predominantly AP-2ε positive. (I) Quantification of GAP43-positive VSNs indicates a significant increase in cKOs compared with controls (*n*=6). Data are mean±s.e.m. (J,K) Ki67 immunostaining and quantification in control (J) and cKO (K). Arrows and arrowheads indicate proliferative cells in marginal and medial regions of VNE, respectively. (L) Increased neurogenesis in the medial zones of the VNE (*n*=6). Data are mean±s.e.m. (M-O) Immunostaining (M,N, arrowheads) and quantification (O) of cleaved caspase 3 in control (M) and cKO (N) shows no change in apoptosis (*n*=4). Data are mean±s.e.m. (P-Q′) Immunostaining for pS6 (gray) and apical VSN marker Meis2 (green) in control (P; higher magnification in P′) and cKO (Q; higher magnification in Q′). (R) Quantification of percentage of pS6-positive apical and basal VSNs (*n*=3). Violin plots indicating the mean percentage of pS6^+^ VSNs/biological replicate. Statistical analysis was carried out using a two-tailed unpaired *t*-test.

Smad4 ablation in immature basal VSNs indicates that a lack of intact *Smad4* gene impairs the formation of functional basal VSNs, proper glomeruli formation and cell homeostasis. However, neuronal activity can partially influence VSN connectivity to mitral cells (Hovis et al., 2012), which complicates determining the level at which Smad4 plays a role in these potentially interconnected phenotypes. We therefore sought to determine whether Smad4-dependent signaling is differentially required at distinct developmental timepoints. We exploited the OMPCre mouse line to conditionally ablate Smad4 in both apical and basal VSNs after they reached maturity. In controls, Smad4 immunolabeling at P60 confirmed expression throughout the epithelium, while we found almost complete Smad4 ablation in both apical and basal territories after OMPCre-mediated recombination (Fig. 6A-C). In the epithelium, the only cells positive for Smad4 were immature neurons, which showed immunoreactivity for GAP43 and not for OMP (data not shown).

Morphological observation of the VNE of OMPCre^+/−^/Smad4^flox/flox^ mice at P60 indicated that the localization of apical and basal VSNs within the VNO was not altered after Smad4 ablation. Furthermore, quantification of cells positive for apical and basal markers (Gαi2 and Gαo) did not show differences in VNO cell composition between control (Smad4^flox/flox^) and OMPCre^+/−^/Smad4^flox/flox^ (Fig. 6D-F). However, we detected increased GAP43 immunoreactivity (Fig. 6G-I) in the central and basal regions of the VNE of the OMPCre^+/−^/Smad4^flox/flox^ mice. This result is consistent with what we observed in AP-2εCre^+/−^/Smad4^flox/flox^ mice. OMPCre^+/−^/Smad4^flox/flox^ mice showed an increase in the number of proliferative (Ki67+) progenitors in central regions of the VNE (Fig. 6J-L), with no changes in apoptosis among the genotypes (Fig. 6M-O). These data indicate no dramatic changes in the VNE after Smad4 ablation in mature VSNs.

### Smad4 ablation in mature VSNs does not compromise neuronal activation after odor exposure

**Fig. 7.**
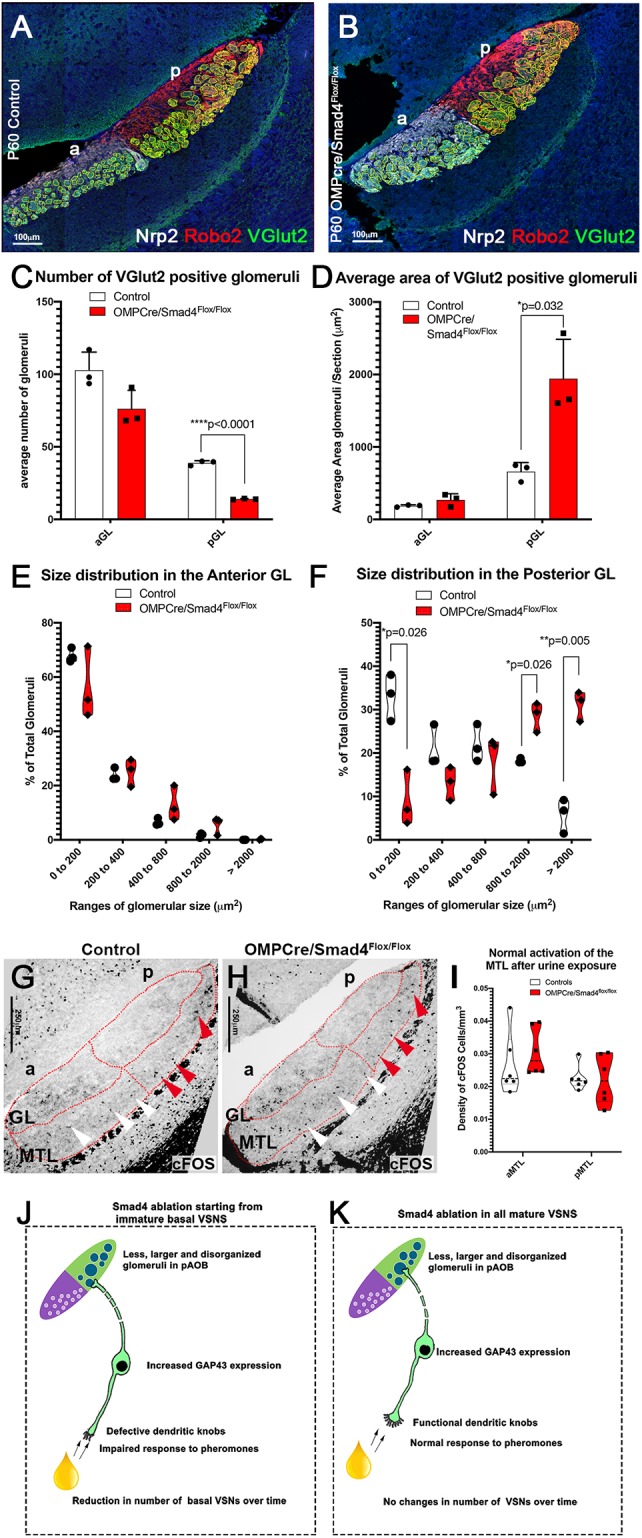
**Basal VSNs form aberrant glomeruli in the pAOB in OMPCre/Smad4^flox/flox^.** (A,B) Immunofluorescence against Nrp2, Robo2 and VGlut2, highlighting fibers projecting to the glomeruli in the aAOB and pAOB. a, anterior; p, posterior. (C) Number of VGlut2^+^ glomeruli in posterior and anterior glomerular regions. Graph shows a decrease in the number of glomeruli in pAOB (*n*=3). Data are mean±s.e.m. (D) The area of VGlut2^+^ glomeruli were quantified. Graph shows an increase in the size of glomeruli in the pAOB (*n*=3). Data are mean±s.e.m. (E,F) Percentage of glomeruli in area ranges was quantified between genotypes for aAOB and pAOB, showing no change in aAOB (E), and a significant reduction in the number of smaller glomeruli and an increase in larger glomeruli in pAOB (F). Violin plot indicating the mean percentage of total glomeruli in size ranges/biological replicate (G-I) Immunostaining (G,H) and quantification (I) of Fos in P60 controls (Smad4^flox/flox^) and P60 OMPCre^+/−^/Smad4^flox/flox^, shows normal activation in MTL in OMPCre/Smad4cKOs (*n*=4). Violin plot indicating the mean of density of Fos cells/biological replicate. (J,K) Cartoon summarizing phenotypes observed in basal VSNs and AOBs of (J) AP-2εCre^+/−^/Smad4^flox/flox^ and (K) OMPCre^+/−^/Smad4^flox/flox^. Statistical analysis was carried out using a two-tailed unpaired *t*-test.

We then determined whether Smad4 is required for VSNs activity in response to olfactory stimuli. We examined VSN activation after whole-male urine exposure by exploiting pS6 as a readout for VSN activation (Silvotti et al., 2018). OMPCre^+/−^/Smad4^flox/flox^ mice showed no difference in the percentage of basal or apical VSN activation compared with controls (Fig. 6P-R). This result suggests that Smad4 ablation, after VSNs have reached maturity, does not compromise the ability of VSNs to respond to stimuli. Next, we performed immunostaining against Fos on AOB sections of controls and OMPCre^+/−^/Smad4^flox/flox^ mice previously exposed to whole-male urine. This analysis did not reveal differences in the functional connectivity of VSNs to the brain among genotypes (Fig. 7G-I). Based on these results, we conclude that mature neurons do not require Smad4 activity for odor detection, synaptic formation and cell homeostasis.

### Ablation of Smad4 in mature VSNs produces a disorganized glomerular layer in only the pAOB

By analyzing VSN projections to the glomeruli in the AOB of OMPCre^+/−^/Smad4^flox/flox^ mice compared with controls, we found a reduced number of glomeruli in the pAOB. Within the pAOB of OMPCre^+/−^/Smad4^flox/flox^ mutants, we found a significant decrease in the percentage of glomeruli with an area less than 200 µm^2^ and an increase in the percentage of glomeruli with an area more than 800 µm^2^ (Fig. 7A-F). However, we did not observe changes in the average glomeruli size in the aAOB in OMPCre^+/−^/Smad4^flox/flox^. These observations indicate that, even as the neurons start to mature (Prince et al., 2009), Smad4 is necessary to form the correct connections of the basal VSNs to the pAOB but not for apical neurons. Moreover, the aberrant glomeruli formation in OMPCre mutants, in which we detected no defects in odor detection and homeostasis, suggest a specific role for Smad4 in defining postsynaptic targeting.

### Loss of Smad4 alters the expression of several surface molecules and presynaptic components

**Fig. 8.**
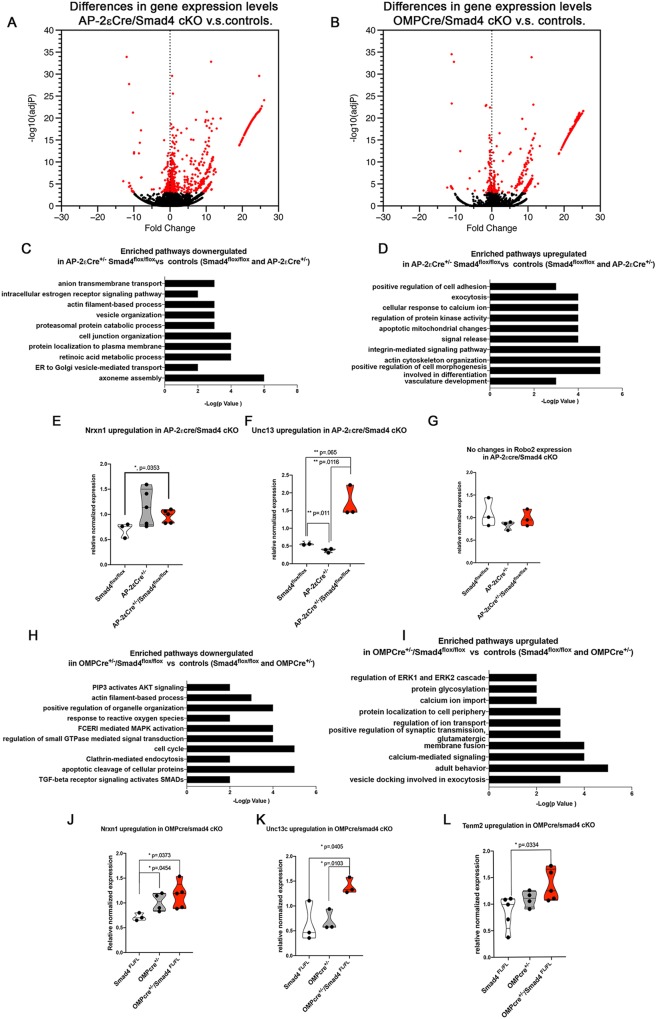
**Transcriptome analysis of Smad4 cKO.** (A,B) Volcano plot highlighting the changes in gene expression in the two cKOs with their respective controls. (C,D) Enriched pathways up- and downregulated in AP-2eSmad4 cKOs. (E-G) qPCR validation indicated upregulation of Nrxn1 and Unc13c, and no changes in Robo2. Violin plot indicating mean of relative normalized expression of transcript/biological replicate. (H,I) Enriched pathway up- and downregulated in OMPCre/Smad4cKO. (J-L) qPCR validation confirmed upregulation of Nrxn1, Unc13c and Tenm2. Violin plot indicating mean of relative normalized expression of transcript/biological replicate. Statistical analysis was carried out using a two-tailed unpaired *t*-test.

To further elucidate the underlying molecular mechanisms that induce aberrant glomeruli formation in Smad4cKOs, we performed mRNA-seq transcriptome analysis of whole VNOs from AP-2εCre^+/−^/Smad4^flox/flox^, OMPCre^+/−^/Smad4^flox/flox^, relative Cre heterozygous control and Smad4^flox/flox^ control (GSE134492) mice. Our data indicated that a lack of functional Smad4 in immature (AP-2εCre^+/−^/Smad4^flox/flox^) basal VSNs induced a significant increase in the expression of genes involved in synapse formation and glutamate release, including Unc13c, synatoptagmin XIII, Dlg2, Adcy3, Plcb2 and Nrxn1. We confirmed the changes in Unc13c and Nrxn1 expression by qRT-PCR (Fig. 8E,F). Notably, a previous study found that the levels of none of these genes was altered in AP-2εCre null mice compared with controls (Lin et al., 2018) (deposited in GEO under accession number GSE110083).

Transcriptome data from OMPCre^+/−^/Smad4^flox/flox^ mice compared with relative controls (GSE134492) also showed significant upregulation of presynaptic molecules, such as those involved in synaptic signaling (Unc13c) and synaptic assembly (Nrxn1) (Fig. 8J-L). These mutants also showed changes in tenurin-m2 (Tenm2), which participates in defining neuronal diversity and glomeruli formation (Berns et al., 2018; Mosca et al., 2012). OMP levels can also alter normal olfactory glomerular mapping (Albeanu et al., 2018). Our qRT-PCR analysis revealed similar changes in Nrxn1 levels between OMPCre^+/−^ and OMPCre^+/−^/Smad4^flox/flox^. These data suggest that at least part of the aberrant Nrxn1 expression observed in the OMPCre conditional KOs stems from only decreased OMP expression (Albeanu et al., 2018). We also observed that OMPCre^+/−^ mice had intermediate, but not significantly different, Tenm2 expression levels between wild-type and OMPCre^+/−^/Smad4^flox/flox^ mice, suggesting that OMP acts as a modifier to modulate Tenm2 levels. However, there was a significant increase in Tenm2 expression levels in OMPCre^+/−^/Smad4^flox/flox^ compared with Smad4^flox/flox^ controls.

The glomerular phenotype of Smad4 mutants resembles the phenotype described after the loss of specific adhesion molecules of the Kirrel family (Brignall et al., 2018; Prince et al., 2013). Our data did not indicate significant changes in mRNA levels for Kirrel family members (deposited in GEO under accession number GSE134492). Gross observation after immunostaining against kirrel 2 and kirrel 3 on both AP-2ε- and OMP-mediated Smad4 conditional KOs did not highlight obvious abnormalities in differential Kirrel expression (Prince et al., 2013). These data suggest that changes in BMP/TGFβ signaling may alter normal expression of genes involved in axonal organization and the glomeruli formation of basal VSN subtypes.

## DISCUSSION

BMPs control proliferation, differentiation, patterning and survival of multiple tissues during embryonic development (Beites et al., 2009; Kuschel et al., 2003; Le Dreau et al., 2012; Liem et al., 2000, 1995; Lim et al., 2000; Schmidt et al., 1995; Tadokoro et al., 2016; Timmer et al., 2002). Our study shows that canonical BMP signaling is necessary for maturation of functional basal VSNs and for their circuit formation with the AOB.

By analyzing the VNO transcriptome, we found and verified that the VNE of mice expresses multiple molecules of the TGFβ family (Fig. 1). BMPs bind with various degrees of affinity to collagens that sustain BMP signaling by binding and activating the ligand molecules (Wang et al., 2008). Immunolabeling against activated/phosphorylated Smad1,5,8 as a readout for active BMP signaling revealed that BMP signal transduction is confined to basal VSNs (Fig. 1G-I) with stronger Smad1,5,8 activation in basal VSNs more proximal to the collagen IV-rich basal lamina (Fig. 1J,K). Moreover, we found high levels of BMP signaling in the marginal zones of the VNO, where VSNs form and begin to mature (Fig. S1). Active BMP signaling in maturing and mature basal VSNs prompted us to elucidate its roles during various contexts/maturation stages.

Using AP-2εCre-mediated Smad4 ablation in maturing basal VSNs, we observed no obvious morphological defects 2 weeks after birth (Fig. 3). However, our 2 months after birth analysis revealed a dramatic reduction in the number of basal VSNs. A slow and progressive reduction of VSNs is a common phenotype in animals with impaired chemosensory detection/signal transduction (Chamero et al., 2011; Montani et al., 2013; Stowers et al., 2002). Histological and functional analysis revealed that AP-2εCre^+/−^/Smad4^flox/flox^ displayed abnormal glomeruli formation in the pAOB and compromised ability of basal VSNs to respond pheromonal stimuli (Figs 4 and 5). In order to understand the mechanism underlying the reduced response to stimuli, we analyzed our transcriptome data and identified changes in the calcium-activated chloride channel (CaCC) TMEM16A, which is a known player in controlling the generation of receptor potential in response to odorants (Fig. 4). Histological analysis of vomeronasal sections from AP-2εCre/Smad4cKO VSNs revealed defects in microvilli and dendritic knob formation, which are not compatible with normal odor detection (Fig. 4P-U). These defects were found in both adult and young animals (Fig. S3C and Fig. 4P-U), where no differences in basal VSNs were found. This suggests that the observed progressive cell reduction is likely secondary to functional loss (Chamero et al., 2011). Notably, in OMPCreSmad4^flox/flox^, where Smad4 was ablated in both mature apical and basal VSNs (Fig. 6), we did not find defects in odor detection (Fig. 6R).

Our results indicating differential effects on neuronal functioning after Smad4 ablation at distinct VSN maturation stages suggest that Smad4 drives genetic programs underlying the functionality of basal VSNs within a critical developmental window. The idea that temporally/context-specific roles for Smad4 signaling are involved in triggering specific aspects of basal VSN maturation is further supported by our finding of no changes in odor detection or expression of knob-specific genes after OMPCre-mediated Smad4 ablation in VSNs at advanced maturation (Fig. 7J,K). Although Smad complexes have weak affinity for DNA (Shi et al., 1998), Smads interact with other site-specific transcription factors to stabilize their DNA binding (Massagué, 2012). Smad-mediated intracellular signaling can regulate transcription by remodeling the chromatin landscape via recruitment of co-activators and/or co-repressors (Ross et al., 2006). Moreover, Smad interaction with other transcription factors integrates signals from the TGFβ family ligands with other signaling cues (Budi et al., 2019). These features add spatial and temporal specificity to active Smad4-mediated signaling with respect to surrounding cellular signals and the maturation stage of VSNs. Future studies will elucidate the role that BMP signaling plays in defining the chromatin landscape of maturing VSNs and how much of our observed phenotype is under direct Smad4-mediated regulation of specific gene targets.

We identified two overlapping phenotypes between AP-2εCre and OMPCre-Smad4cKOs: increased GAP43 expression and the formation of larger disorganized glomeruli in the pAOB. These phenotypes in OMPCre-Smad4cKOs suggest that Smad4 intracellular signaling, irrespective of the VSN maturation stage and sensory ability, plays a role in controlling gene expression that regulates axonal identity and connectivity of basal VSNs to the brain. Notably, our conclusion does seem true only for basal neurons. Based on our results, we posit that distinct molecular mechanisms control the development and function of the apical and basal VSNs.

Pheromones present in whole-male urine activate both apical and basal VSNs and therefore anterior and pAOBs. In order to understand whether the VSN are still able to respond to pheromonal stimulus in the mutants, we analyzed the neuronal activation in response to whole-male urine in AP-2εCre^+/−/^Smad4^flox/flox^ mutants and found an unexpected reduction in the number of Fos-expressing mitral cells in the aAOB (Fig. 5L). Although the reduction of Fos activation in the pAOB likely reflects odor detection defects secondary to Smad4 ablation in the basal VSNs (Fig. 4), we posit the reduction in Fos activation in the aAOB reflects a postsynaptic/mitral cell defect. This speculation is consistent with our previous report that mitral cells of the aAOB, but not the pAOB, are positive for the AP-2ε lineage (Lin et al., 2018). The reduced number of Fos-expressing mitral cells in the aAOB of AP-2εCre/Smad4 mutants suggests Smad4 contributes to controlling maturation or functioning of anterior mitral cells.

Even though the aberrant glomerular morphology we observed in Smad4 mutants resembles that described in Kirrel 2,3 knockout mice (Prince et al., 2013; Brignall et al., 2018), our transcriptome analyses of Smad4cKOS and relative Cre controls did not reveal expression changes for Kirrel gene mRNA. Rather, we found upregulation of several presynaptic genes, including *Nrxn1* and *Unc13c* (see Table S3). Nrxn1 is a transmembrane synaptic adhesion molecule that regulates synaptic architecture and function in the brain. The interaction between trans-synaptic molecules, such as Nrxn1 and Ncam1, is crucial for defining pre- and postsynaptic target recognition alignment during synaptogenesis and synaptic transmission (Gerrow and El-Husseini, 2006; Scheiffele, 2003; Yamagata et al., 2003). Future studies can elucidate whether changes in Nrxn1 expression (Ko et al., 2009; Südhof, 2017) can define synaptic identity, and pre- and postsynaptic compatibility in the glomerulus.

The spatial position of stem cells in the olfactory epithelium determines the identity of olfactory neurons (Coleman et al., 2019). Our data suggest that basal VSNs receive different levels of BMP signaling based on their distance from the basal lamina (Fig. 1). Based on our results, we speculate that the relative position of VSNs with respect to morphogenic signals in the VNE play a determining factor in tuning the expression levels of specific genes, such as glycoproteins, adhesion molecules and presynaptic components, that define axonal identity, targeting specificity and synaptic compatibility. Moreover cell bodies of VSNs expressing different V2Rs have been reported to reside in different sublayers of the basal territory of the VNO (Ishii and Mombaerts, 2008). Whether specific relationships exist between VR expression, the type and level of adhesion molecules expressed, cell body position and Smad4 signaling is worth further investigation. For both Smad4cKO models, we found gene expression changes in various synaptic and surface molecules, including Nrxn1. In addition, OMPCre/Smad4cKOs showed significant upregulation of the glycoprotein Tenm2, a molecule crucial for defining synaptic matching and glomeruli formation in *Drosophila* olfactory neurons (Hong et al., 2012). Additional studies using more-sensitive methods such as single cell RNAseq can delineate how altering morphogenic signal gradients specifically modulates axonal identity and targeting of specific neuronal types.

Retrograde BMP signaling in neurons can control synaptic growth at neuromuscular junctions, and define neuronal identity and somatosensory map formation of the trigeminal nerve in rodents (Ball et al., 2010; Banerjee and Riordan, 2018; Berke et al., 2013; Fuentes-Medel and Budnik, 2010; Hegarty et al., 2013; Hodge et al., 2007; Liao et al., 2018; Piccioli and Littleton, 2014). The experimental approach used here does not permit the dissociation of aberrant local vomeronasal BMP signaling and putative retrograde BMP signaling from the AOB. However, we feel these alternative findings are unlikely, as we found similar patterns in p-Smad1/5/8 in VNE of Arx-1 null mice, which is a mouse line that lacks olfactory bulbs and has little or no connections between the VNO and the brain (Taroc et al., 2017).

Overall, this study reveals that compromising Smad4-mediated signaling in maturing basal VSNs is not compatible with the formation of functional basal VSNs, normal glomeruli formation and survival. Smad4 loss of function in mature VSNs does not affect odor detection in the VNE or VSN survival but still disrupts normal glomeruli formation.

TGFβ and BMP inhibitors might be a novel class of candidate pharmacological targets for treatment of several human pathologies, including fibrosis and cancers (Huynh et al., 2019; Ramzy et al., 2018; Soji et al., 2018). However, our data on VSNs suggest caution until we have a detailed analysis of the broad effects of TGF-β/Smad inhibitors on homeostasis and connectivity of all nervous system neurons at different maturation stages.

## MATERIALS AND METHODS

### Animals

AP-2εCre line [TfAP-2e^tm1(cre)Will^] was donated by Dr Trevor Williams (University of Colorado, USA). The OMPCre line was donated by Dr Paul Feinstein (Hunter College, City University of New York, USA). Smad4^flox/flox^ (*Smad4^tm2.1Cxd^*/J, stock 017462) and R26RYFP [B6.129X1-Gt(*ROSA*)26Sor*^tm1(EYFP)Cos^*/J, stock 00614] were purchased from Jackson Laboratories. Experimental analyses were carried out in offspring homozygous for floxed Smad4 and AP-2εCre^+/−^ (AP-2εCre^+/−^/Smad4^flox/flox^ or AP-2εCre^+/−^/Smad4cKO) or Smad4^flox/flox^ and OMPCre^+/−^ (OMPCre^+/−^/Smad4^flox/flox^ or OMPCre^+/−^/Smad4cKO), referred to as conditional Smad4 mutants. The controls used were Smad4^flox/flox^, unless otherwise indicated. These mice appeared healthy and survived to adulthood. Mice of either sex were used for *in situ* hybridization and immunohistochemistry experiments. All experiments involving mice were approved by the University at Albany Institutional Animal Care and Use Committee (IACUC).

### Tissue preparation

Tissue was collected after transcardial perfusion with PBS then 3.7% formaldehyde in PBS. Mouse brains were then immersion fixed in 1% formaldehyde at 4°C overnight. Noses were immersion fixed in 3.7% formaldehyde in PBS at 4°C overnight and decalcified in 500 mM EDTA for 3-7 days. After fixation and decalcification, samples were cryoprotected in 30% sucrose overnight at 4°C, then embedded in OCT (Tissue-TeK) and stored at −80°C. Samples were cryosectioned using CM3050S Leica cryostat at 16 μm (nose) and 20 μm (brain) collected on Superfrost Plus Micro Slides (VWR). All sections were stored at −80°C until ready for staining or *in situ* hybridization.

### Immunohistochemistry

Primary antibodies and concentrations were *Gt α-AP-2ε (2 µg/ml, sc-131393 X, Santa Cruz), *Rb α-cleaved caspase-3 (1:1000, AB3623, Millipore), *Ms α-Gαi2 (1:200, 05-1403, Millipore), Rb α-Gαo (1:1000, 551, Millipore), Rb α-GAP43 (1:500, 16053, Abcam), *Rb α-Ki67 (1:1000, AB9260, Millipore), *Gt α-Kirrel2 (1:500, AF52930, R&D Systems), *Ms α-Kirrel3 (1:100, 75-333, NeuroMab), Gt α-NPN2 (1:4000, AF567, R&D Systems), Gt α-OMP (1:4000, 5441001, WAKO), *Ms α-Robo2 (1:100, 376177, Santa Cruz), Rb α-V2R2 (1:4000, a gift from Dr Roberto Tirindelli, Universitá di Parma, Italy), *Rb α-pSmad 2 (1:800, AB 3849, Millipore), *Rb α-pSmad1,5,8 (1:50, AB3848-I, Millipore), Rb α-Smad4 (1:200, AB40759, Abcam), Chk α-VGlut2(1:500, 135416, Synaptic Systems), *Rbα-pS6 (ser 240/244) (1:500, D68F8, Cell Signaling Technology), *Rbα-Fos (1:250, Cell Signaling Technology), *Rbα-Villin (1:2000, Abcam, ab130751) and Rbα-Tmem16a (1:50, Abcam, ab53212). Microwave antigen retrieval in citrate buffer pH 6 was performed for the indicated antibodies (*) (Forni et al., 2006). All primary antibodies were incubated at 4°C overnight.

For immunoperoxidase staining procedures, slides were processed using standard protocols (Forni et al., 2013) and staining was visualized (Vectastain ABC Kit) using diaminobenzidine (DAB) in a glucose solution containing glucose oxidase to generate hydrogen peroxide. After immunostaining, sections were counterstained with Methyl Green. For immunofluorescence, species-appropriate secondary antibodies were conjugated with Alexa-488, Alexa-594 or Alexa-568 (Molecular Probes). Immunofluorescent sections were counterstained with 4′,6′-diamidino-2-phenylindole (DAPI, 1:3000; Sigma-Aldrich) and mounted with FluoroGel (Electron Microscopy Services). Confocal microscopy pictures were taken on a Zeiss LSM 710 microscope. Bright-field and epifluorescence pictures were taken on a Leica DM4000 B LED fluorescence microscope attached to Leica DFC310 FX camera. Images were analyzed and quantified using FIJI/ImageJ software.

### *In situ* hybridization

Digoxigenin-labeled cRNA probes against Smad4 Exon 8, BMP4 and BMP6 were prepared by *in vitro* transcription (DIG RNA labeling kit; Roche Diagnostics). Plasmids to generate cRNA probes for Smad4 and for BMP4 and BMP6 were gifts from Prof. Dr Rolf Zeller (University of Basel, Switzerland) and Dr Kapil Bharti (Ocular and Stem Cell Translational Research Unit, National Eye Institute, Bethesda, MD, USA), respectively. *In situ* hybridization was performed as described previously (Forni et al., 2013) and visualized by immunostaining with an alkaline phosphatase-conjugated anti-DIG (1:1000) and NBT/BCIP developer solution (Roche Diagnostics).

### Whole-male urine exposure

Two-month-old male Smad4^flox/flox^ control and conditional KO mice were single caged 2-3 days before the experiment. Male urine was collected and pooled from mice of mixed backgrounds. Male control and experimental mice were exposed to whole-male urine, then perfused and collected 90 min after exposure. The brains were sectioned parasagittally and the noses were sectioned coronally, then immunostained for Fos and pS6, respectively. BS-Lectin I conjugated to Rhodamine (Vector) was used in order to distinguish between anterior and pAOB (Prince et al., 2013).

### Quantification and statistical analyses of microscopy data

Data were collected from mice kept in the same housing conditions. Measurements of areas, thickness and cell counts were performed on digital pictures using FIJI/ImageJ. For some of the antigens that have different expression from the tips of the VNO to the center of the epithelium (where cells are mostly mature neurons), cell counts were made by overlapping a mask to equally divide the VNO into seven 36° angled zones (de la Rosa-Prieto et al., 2010).

For all AOB quantifications, the most medial three parasagittal sections per AOB were used. We used confocal images of triple staining against VGlut2, Npn2 and Robo2 to trace the VGlut2-positive glomeruli in both Npn2-positive aAOB and Robo2-positive pAOB. Using the Fiji imaging tool, we generated maximum intensity projection images from the 3D stack to increase signal-to-noise ratio and manually traced each glomerulus. We analyzed the average number and glomeruli area in anterior and pAOB for each section. This was then used to determine the overall average of all technical replicates from each biological replicate. Number of glomeruli within area ranges were calculated and then compared between genotypes.

In P15 and older animals, the most medial six to eight VNE sections were quantified for each series and averaged. Data from each genotype were grouped and used for statistical analysis. Statistical analysis was performed using GraphPad, Prism 7.0b. A two-tailed unpaired *t*-test was used for statistical analyses and *P*<0.05 was considered statistically significant. Sample sizes and *P*-values are indicated in figure legends or in each graph.

### Analysis of the dendritic knobs using Imaris

Confocal images from OMP, GFP and villin triple immunostaining on genetically lineage traced AP-2εCreRYFP control and AP-2εCre Smad4^Flox/Flox^ RYFP were analyzed using Imaris software (version 9.5, Bitplane). To quantify Tmem16a expression levels in basal knobs, we selected the YFP channel as the input for spots object creation. Objects were generated using background subtraction with thresholding automatically calculated in Imaris. We then isolated only spot objects corresponding to basal knobs and recorded the mean intensity of Tmem16a immunofluorescence of all the voxels contained in each spot. These values were used to calculate the average TMEM16a intensity of basal dendritic knobs, three or four sections/VNO were quantified. To quantify OMP, villin and YFP knobs, we selected the channel corresponding to villin to extract the apical region of the epithelium where the knobs reside. Then, to identify the knobs, we set a colocalization threshold between OMP and villin (the mean intensity of OMP and villin within the region of interest). We then built a new colocalization channel to generate the objects corresponding to the knobs. Spot objects were filtered to discriminate between YFP-positive and YFP-negative knobs using an intensity of at least half the maximum mean intensity of all the knobs. We used the average of the cutoff values of the controls as a parameter to identify comparable knobs in conditional KOs (see Movies 1 and 2). This method of quantification provided the total number of knobs (OMP+; Villin+) and the number of YFP-positive knobs according to our cutoff. Based on these results, we calculated the percentage of ‘normal’ basal knobs in control and cKO.

### RT-PCR

cDNA was synthesized from 1000 ng of RNA extracted from whole VNO of P21 Smad4^flox/flox^ animals (*n*=3) using the Super ScriptIII First Strand kit (Invitrogen, 18080400). RT-PCR for BMP2, BMP3, BMP4, BMP6, BMP7, TGFβ1, TGFβ2 and GDF10 was carried out using cDNA generated from RNA isolated from dissected VNO of Smad4^flox/flox^ mice using the primers indicated in Table S1.

### qPCR

cDNA was synthesized from 500 ng of RNA extracted from whole VNO of control animals (P21) (Smad4^flox/flox^ and AP-2εCre+/− or Smad4^flox/flox^ and OMPCre+/−) and cKOs (P21) (AP-2εCre+/−;Smad4^flox/flox^ or OMPCre+/−;Smad4^flox/flox^) using the Invitrogen Super Script III First Strand kit. The expression of Nrxn1, Unc13c, Tenm2 and Robo2 was validated by qRT PCR using primers listed in Table S2. Three technical replicates of each sample were run and a standard curve was used to get relative quantities, which were then normalized to ubiquitin C.

### Transcriptome analysis

Total RNA was isolated using PureLink RNA mini kit (12183018A) from dissected VNO of control (Smad4^flox/flox^, AP-2εCre^+/−^ and OMPCre^+/−^) and Smad4cKOs (AP-2εCre^+/−^/Smad4^flox/flox^ and OMPCre^+/−^/Smad4^flox/Flox^). RNA with a RIN number higher than 8 on an Agilent Bioanalyzer was used for further experiments. Libraries for mRNA-seq were prepared using NEXTflex Rapid directional mRNA-seq Bundle (5138-10). To prepare libraries, PolyA^+^ RNA was isolated from 1 µg of total RNA using two rounds of selection with poly dT magnetic beads (BioO Scientific) and used as the template for first strand synthesis using random hexamers. Second-strand synthesis was performed using the standard RNAseH-mediated nicking with dUTP for preserving strandedness. Double-stranded cDNA was then used as the template for library construction following the manufacturer's recommendations for 1 µg of starting total RNA (BioO Scientific NextFlex). Resulting libraries were quantified using the NEBNext Library Quant Kit for Illumina (New England Biolabs) and sizes were confirmed using an Agilent Bioanalyzer. Libraries were sequenced on an Illumina NextSeq 500 instrument for 75 cycles in single-read orientation. The resulting raw sequencing files were quantified using salmon (v 0.14.0) in quant mode and the validateMappings flag using the ENSMBL mm10 GRC38 mouse CDS assembly. Raw read counts per gene were imported into R and differential gene expression was determined using DESeq2 (v 1.22.2) (Love et al., 2014). Gene Ontology was performed using the MetaScape package (Zhou et al., 2019). The transcriptome data are available in GEO under accession number GSE134492.

## Supplementary Material

Supplementary information

Reviewer comments

## References

[DEV184036C1] Albeanu D. F., Provost A. C., Agarwal P., Soucy E. R., Zak J. D. and Murthy V. N. (2018). Olfactory marker protein (OMP) regulates formation and refinement of the olfactory glomerular map. *Nat. Commun.* 9, 5073 10.1038/s41467-018-07544-9 30498219 PMC6265328

[DEV184036C2] Ball R. W., Warren-Paquin M., Tsurudome K., Liao E. H., Elazzouzi F., Cavanagh C., An B.-S., Wang T.-T., White J. H. and Haghighi A. P. (2010). Retrograde BMP signaling controls synaptic growth at the NMJ by regulating trio expression in motor neurons. *Neuron* 66, 536-549. 10.1016/j.neuron.2010.04.011 20510858

[DEV184036C3] Banerjee S. and Riordan M. (2018). Coordinated regulation of axonal microtubule organization and transport by Drosophila Neurexin and BMP pathway. *Sci. Rep.* 8, 17337 10.1038/s41598-018-35618-7 30478335 PMC6255869

[DEV184036C4] Beites C. L., Kawauchi S. and Calof A. L. (2009). Olfactory neuron patterning and specification. *Dev. Neurobiol.* 7, 145-156. 10.1016/B978-008045046-9.01046-9 24817923 PMC4015351

[DEV184036C5] Belluscio L., Koentges G., Axel R. and Dulac C. (1999). A map of pheromone receptor activation in the mammalian brain. *Cell* 97, 209-220. 10.1016/S0092-8674(00)80731-X 10219242

[DEV184036C6] Benazet J.-D., Pignatti E., Nugent A., Unal E., Laurent F. and Zeller R. (2012). Smad4 is required to induce digit ray primordia and to initiate the aggregation and differentiation of chondrogenic progenitors in mouse limb buds. *Development* 139, 4250-4260. 10.1242/dev.084822 23034633

[DEV184036C7] Berke B., Wittnam J., McNeill E., Van Vactor D. L. and Keshishian H. (2013). Retrograde BMP signaling at the synapse: a permissive signal for synapse maturation and activity-dependent plasticity. *J. Neurosci.* 33, 17937-17950. 10.1523/JNEUROSCI.6075-11.2013 24198381 PMC3818560

[DEV184036C8] Berns D. S., DeNardo L. A., Pederick D. T. and Luo L. (2018). Teneurin-3 controls topographic circuit assembly in the hippocampus. *Nature* 554, 328-333. 10.1038/nature25463 29414938 PMC7282895

[DEV184036C9] Brann J. H. and Firestein S. (2010). Regeneration of new neurons is preserved in aged vomeronasal epithelia. *J. Neurosci.* 30, 15686-15694. 10.1523/JNEUROSCI.4316-10.2010 21084624 PMC3393108

[DEV184036C10] Brignall A. C. and Cloutier J.-F. (2015). Neural map formation and sensory coding in the vomeronasal system. *Cell. Mol. Life Sci.* 72, 4697-4709. 10.1007/s00018-015-2029-5 26329476 PMC11113928

[DEV184036C11] Brignall A. C., Raja R., Phen A., Prince J. E. A., Dumontier E. and Cloutier J.-F. (2018). Loss of Kirrel family members alters glomerular structure and synapse numbers in the accessory olfactory bulb. *Brain Struct. Funct.* 223, 307-319. 10.1007/s00429-017-1485-0 28815295

[DEV184036C12] Budi E. H., Hoffman S., Gao S., Zhang Y. E. and Derynck R. (2019). Integration of TGF-beta-induced Smad signaling in the insulin-induced transcriptional response in endothelial cells. *Sci. Rep.* 9, 16992 10.1038/s41598-019-53490-x 31740700 PMC6861289

[DEV184036C13] Bunt S., Hooley C., Hu N., Scahill C., Weavers H. and Skaer H. (2010). Hemocyte-secreted type IV collagen enhances BMP signaling to guide renal tubule morphogenesis in Drosophila. *Dev. Cell* 19, 296-306. 10.1016/j.devcel.2010.07.019 20708591 PMC2941037

[DEV184036C14] Chamero P., Katsoulidou V., Hendrix P., Bufe B., Roberts R., Matsunami H., Abramowitz J., Birnbaumer L., Zufall F. and Leinders-Zufall T. (2011). G protein G(alpha)o is essential for vomeronasal function and aggressive behavior in mice. *Proc. Natl. Acad. Sci. USA* 108, 12898-12903. 10.1073/pnas.1107770108 21768373 PMC3150917

[DEV184036C15] Chamero P., Leinders-Zufall T. and Zufall F. (2012). From genes to social communication: molecular sensing by the vomeronasal organ. *Trends Neurosci.* 35, 597-606. 10.1016/j.tins.2012.04.011 22658923

[DEV184036C16] Cloutier J.-F., Giger R. J., Koentges G., Dulac C., Kolodkin A. L. and Ginty D. D. (2002). Neuropilin-2 mediates axonal fasciculation, zonal segregation, but not axonal convergence, of primary accessory olfactory neurons. *Neuron* 33, 877-892. 10.1016/S0896-6273(02)00635-9 11906695

[DEV184036C17] Coleman J. H., Lin B., Louie J. D., Peterson J., Lane R. P. and Schwob J. E. (2019). Spatial determination of neuronal diversification in the olfactory epithelium. *J. Neurosci.* 39, 814-832. 10.1523/JNEUROSCI.3594-17.2018 30530861 PMC6382982

[DEV184036C18] de la Rosa-Prieto C., Saiz-Sanchez D., Ubeda-Bañon I., Argandoña-Palacios L., Garcia-Muñozguren S. and Martinez-Marcos A. (2010). Neurogenesis in subclasses of vomeronasal sensory neurons in adult mice. *Dev. Neurobiol.* 70, 961-970. 10.1002/dneu.20838 20848614

[DEV184036C19] Del Punta K., Puche A., Adams N. C., Rodriguez I. and Mombaerts P. (2002). A divergent pattern of sensory axonal projections is rendered convergent by second-order neurons in the accessory olfactory bulb. *Neuron* 35, 1057-1066. 10.1016/S0896-6273(02)00904-2 12354396

[DEV184036C20] Dulac C. (2000). Sensory coding of pheromone signals in mammals. *Curr. Opin. Neurobiol.* 10, 511-518. 10.1016/S0959-4388(00)00121-5 10981622

[DEV184036C21] Enomoto T., Ohmoto M., Iwata T., Uno A., Saitou M., Yamaguchi T., Kominami R., Matsumoto I. and Hirota J. (2011). Bcl11b/Ctip2 controls the differentiation of vomeronasal sensory neurons in mice. *J. Neurosci.* 31, 10159-10173. 10.1523/JNEUROSCI.1245-11.2011 21752992 PMC3394408

[DEV184036C22] Forni P. E., Scuoppo C., Imayoshi I., Taulli R., Dastru W., Sala V., Betz U. A., Muzzi P., Martinuzzi D., Vercelli A. E. et al. (2006). High levels of Cre expression in neuronal progenitors cause defects in brain development leading to microencephaly and hydrocephaly. *J. Neurosci.* 26, 9593-9602. 10.1523/JNEUROSCI.2815-06.2006 16971543 PMC6674592

[DEV184036C23] Forni P. E., Bharti K., Flannery E. M., Shimogori T. and Wray S. (2013). The indirect role of fibroblast growth factor-8 in defining neurogenic niches of the olfactory/GnRH systems. *J. Neurosci.* 33, 19620-19634. 10.1523/JNEUROSCI.3238-13.2013 24336726 PMC3858631

[DEV184036C24] Francia S., Pifferi S., Menini A. and Tirindelli R. (2014). Vomeronasal receptors and signal transduction in the vomeronasal organ of mammals. In *Neurobiology of Chemical Communication* (ed. Mucignat-Caretta C.), Chapter 10. Boca Raton, FL https://www.ncbi.nlm.nih.gov/pubmed/24830038 24830038

[DEV184036C25] Fuentes-Medel Y. and Budnik V. (2010). Ménage à Trio during BMP-mediated retrograde signaling at the NMJ. *Neuron* 66, 473-475. 10.1016/j.neuron.2010.05.016 20510851 PMC3499985

[DEV184036C26] Garamszegi N., Garamszegi S. P., Samavarchi-Tehrani P., Walford E., Schneiderbauer M. M., Wrana J. L. and Scully S. P. (2010). Extracellular matrix-induced transforming growth factor-β receptor signaling dynamics. *Oncogene* 29, 2368-2380. 10.1038/onc.2009.514 20101206

[DEV184036C82] Gerrow K., Romorini S., Nabi S. M., Colicos M. A., Sala C. and El-Husseini A. (2006). A preformed complex of postsynaptic proteins is involved in excitatory synapse development. *Neuron* 49, 547-562. 10.1016/j.neuron.2006.01.015 16476664

[DEV184036C27] Giacobini P., Benedetto A., Tirindelli R. and Fasolo A. (2000). Proliferation and migration of receptor neurons in the vomeronasal organ of the adult mouse. *Brain Res. Dev. Brain Res.* 123, 33-40. 10.1016/S0165-3806(00)00080-8 11020548

[DEV184036C28] Hegarty S. V., O'Keeffe G. W. and Sullivan A. M. (2013). BMP-Smad 1/5/8 signalling in the development of the nervous system. *Prog. Neurobiol.* 109, 28-41. 10.1016/j.pneurobio.2013.07.002 23891815

[DEV184036C29] Hill C. S. (2016). Transcriptional control by the SMADs. *Csh Perspect. Biol.* 8, a022079 10.1101/cshperspect.a022079 PMC5046698 27449814

[DEV184036C30] Hodge L. K., Klassen M. P., Han B.-X., Yiu G., Hurrell J., Howell A., Rousseau G., Lemaigre F., Tessier-Lavigne M. and Wang F. (2007). Retrograde BMP signaling regulates trigeminal sensory neuron identities and the formation of precise face maps. *Neuron* 55, 572-586. 10.1016/j.neuron.2007.07.010 17698011

[DEV184036C31] Höfer D., Shin D. W. and Drenckhahn D. (2000). Identification of cytoskeletal markers for the different microvilli and cell types of the rat vomeronasal sensory epithelium. *J. Neurocytol.* 29, 147-156. 10.1023/A:1026548020851 11428046

[DEV184036C32] Hong W., Mosca T. J. and Luo L. (2012). Teneurins instruct synaptic partner matching in an olfactory map. *Nature* 484, 201-207. 10.1038/nature10926 22425994 PMC3345284

[DEV184036C33] Hovis K. R., Ramnath R., Dahlen J. E., Romanova A. L., LaRocca G., Bier M. E. and Urban N. N. (2012). Activity regulates functional connectivity from the vomeronasal organ to the accessory olfactory bulb. *J. Neurosci.* 32, 7907-7916. 10.1523/JNEUROSCI.2399-11.2012 22674266 PMC3483887

[DEV184036C34] Huynh L. K., Hipolito C. J. and Ten Dijke P. (2019). A perspective on the development of TGF-β inhibitors for cancer treatment. *Biomolecules* 9, 743 10.3390/biom9110743 PMC6921009 31744193

[DEV184036C35] Ishii T. and Mombaerts P. (2008). Expression of nonclassical class I major histocompatibility genes defines a tripartite organization of the mouse vomeronasal system. *J. Neurosci.* 28, 2332-2341. 10.1523/JNEUROSCI.4807-07.2008 18322080 PMC6671199

[DEV184036C36] Isogai Y., Si S., Pont-Lezica L., Tan T., Kapoor V., Murthy V. N. and Dulac C. (2011). Molecular organization of vomeronasal chemoreception. *Nature* 478, 241-245. 10.1038/nature10437 21937988 PMC3192931

[DEV184036C37] Ji S.-J. and Jaffrey S. R. (2012). Intra-axonal translation of SMAD1/5/8 mediates retrograde regulation of trigeminal ganglia subtype specification. *Neuron* 74, 95-107. 10.1016/j.neuron.2012.02.022 22500633 PMC3328135

[DEV184036C38] Ko J., Zhang C., Arac D., Boucard A. A., Brunger A. T. and Südhof T. C. (2009). Neuroligin-1 performs neurexin-dependent and neurexin-independent functions in synapse validation. *EMBO J.* 28, 3244-3255. 10.1038/emboj.2009.249 19730411 PMC2771087

[DEV184036C39] Kumar A., Dudley C. A. and Moss R. L. (1999). Functional dichotomy within the vomeronasal system: distinct zones of neuronal activity in the accessory olfactory bulb correlate with sex-specific behaviors. *J. Neurosci.* 19, RC32 10.1523/JNEUROSCI.19-20-j0003.1999 10516334 PMC6782749

[DEV184036C40] Kuschel S., Rüther U. and Theil T. (2003). A disrupted balance between Bmp/Wnt and Fgf signaling underlies the ventralization of the Gli3 mutant telencephalon. *Dev. Biol.* 260, 484-495. 10.1016/S0012-1606(03)00252-5 12921747

[DEV184036C41] Le Dreau G., Garcia-Campmany L., Rabadan M. A., Ferronha T., Tozer S., Briscoe J. and Marti E. (2012). Canonical BMP7 activity is required for the generation of discrete neuronal populations in the dorsal spinal cord. *Development* 139, 259-268. 10.1242/dev.074948 22159578 PMC3243093

[DEV184036C42] Lee K. J., Dietrich P. and Jessell T. M. (2000). Genetic ablation reveals that the roof plate is essential for dorsal interneuron specification. *Nature* 403, 734-740. 10.1038/35001507 10693795

[DEV184036C43] Liao E. H., Gray L., Tsurudome K., El-Mounzer W., Elazzouzi F., Baim C., Farzin S., Calderon M. R., Kauwe G. and Haghighi A. P. (2018). Kinesin Khc-73/KIF13B modulates retrograde BMP signaling by influencing endosomal dynamics at the Drosophila neuromuscular junction. *PLoS Genet.* 14, e1007184 10.1371/journal.pgen.1007184 29373576 PMC5802963

[DEV184036C44] Liem K. F. Jr., Tremml G., Roelink H. and Jessell T. M. (1995). Dorsal differentiation of neural plate cells induced by BMP-mediated signals from epidermal ectoderm. *Cell* 82, 969-979. 10.1016/0092-8674(95)90276-7 7553857

[DEV184036C45] Liem K. F. Jr., Jessell T. M. and Briscoe J. (2000). Regulation of the neural patterning activity of sonic hedgehog by secreted BMP inhibitors expressed by notochord and somites. *Development* 127, 4855-4866. 11044400 10.1242/dev.127.22.4855

[DEV184036C46] Lim D. A., Tramontin A. D., Trevejo J. M., Herrera D. G., García-Verdugo J. M. and Alvarez-Buylla A. (2000). Noggin antagonizes BMP signaling to create a niche for adult neurogenesis. *Neuron* 28, 713-726. 10.1016/S0896-6273(00)00148-3 11163261

[DEV184036C47] Lin J. M., Taroc E. Z. M., Frias J. A., Prasad A., Catizone A. N., Sammons M. A. and Forni P. E. (2018). The transcription factor Tfap2e/AP-2ε plays a pivotal role in maintaining the identity of basal vomeronasal sensory neurons. *Dev. Biol.* 441, 67-82. 10.1016/j.ydbio.2018.06.007 29928868

[DEV184036C48] Love M. I., Huber W. and Anders S. (2014). Moderated estimation of fold change and dispersion for RNA-seq data with DESeq2. *Genome Biol.* 15, 550 10.1186/s13059-014-0550-8 25516281 PMC4302049

[DEV184036C49] Massagué J. (2012). TGFβ signalling in context. *Nat. Rev. Mol. Cell Biol.* 13, 616-630. 10.1038/nrm3434 22992590 PMC4027049

[DEV184036C50] Mombaerts P., Wang F., Dulac C., Chao S. K., Nemes A., Mendelsohn M., Edmondson J. and Axel R. (1996). Visualizing an olfactory sensory map. *Cell* 87, 675-686. 10.1016/S0092-8674(00)81387-2 8929536

[DEV184036C51] Montani G., Tonelli S., Sanghez V., Ferrari P. F., Palanza P., Zimmer A. and Tirindelli R. (2013). Aggressive behaviour and physiological responses to pheromones are strongly impaired in mice deficient for the olfactory G-protein γ-subunit Gγ8. *J. Physiol.* 591, 3949-3962. 10.1113/jphysiol.2012.247528 23836683 PMC3764639

[DEV184036C52] Mosca T. J., Hong W., Dani V. S., Favaloro V. and Luo L. (2012). Trans-synaptic Teneurin signalling in neuromuscular synapse organization and target choice. *Nature* 484, 237-241. 10.1038/nature10923 22426000 PMC3326183

[DEV184036C53] Münch J., Billig G., Hübner C. A., Leinders-Zufall T., Zufall F. and Jentsch T. J. (2018). Ca(2+)-activated Cl(−) currents in the murine vomeronasal organ enhance neuronal spiking but are dispensable for male-male aggression. *J. Biol. Chem.* 293, 10392-10403. 10.1074/jbc.RA118.003153 29769308 PMC6028972

[DEV184036C54] Paralkar V. M., Vukicevic S. and Reddi A. H. (1991). Transforming growth factor β type 1 binds to collagen IV of basement membrane matrix: implications for development. *Dev. Biol.* 143, 303-308. 10.1016/0012-1606(91)90081-D 1991553

[DEV184036C55] Paralkar V. M., Weeks B. S., Yu Y. M., Kleinman H. K. and Reddi A. H. (1992). Recombinant human bone morphogenetic protein 2B stimulates PC12 cell differentiation: potentiation and binding to type IV collagen. *J. Cell Biol.* 119, 1721-1728. 10.1083/jcb.119.6.1721 1469059 PMC2289768

[DEV184036C56] Piccioli Z. D. and Littleton J. T. (2014). Retrograde BMP signaling modulates rapid activity-dependent synaptic growth via presynaptic LIM kinase regulation of cofilin. *J. Neurosci.* 34, 4371-4381. 10.1523/JNEUROSCI.4943-13.2014 24647957 PMC3960475

[DEV184036C57] Prince J. E. A., Cho J. H., Dumontier E., Andrews W., Cutforth T., Tessier-Lavigne M., Parnavelas J. and Cloutier J.-F. (2009). Robo-2 controls the segregation of a portion of basal vomeronasal sensory neuron axons to the posterior region of the accessory olfactory bulb. *J. Neurosci.* 29, 14211-14222. 10.1523/JNEUROSCI.3948-09.2009 19906969 PMC2821732

[DEV184036C58] Prince J. E. A., Brignall A. C., Cutforth T., Shen K. and Cloutier J.-F. (2013). Kirrel3 is required for the coalescence of vomeronasal sensory neuron axons into glomeruli and for male-male aggression. *Development* 140, 2398-2408. 10.1242/dev.087262 23637329 PMC3653560

[DEV184036C59] Ramzy M. M., Abdelghany H. M., Zenhom N. M. and El-Tahawy N. F. (2018). Effect of histone deacetylase inhibitor on epithelial-mesenchymal transition of liver fibrosis. *IUBMB Life* 70, 511-518. 10.1002/iub.1742 29601129

[DEV184036C60] Ressler K. J., Sullivan S. L. and Buck L. B. (1994). Information coding in the olfactory system: evidence for a stereotyped and highly organized epitope map in the olfactory bulb. *Cell* 79, 1245-1255. 10.1016/0092-8674(94)90015-9 7528109

[DEV184036C61] Roos J., Roos M., Schaeffer C. and Aron C. (1988). Sexual differences in the development of accessory olfactory bulbs in the rat. *J. Comp. Neurol.* 270, 121-131. 10.1002/cne.902700110 3372734

[DEV184036C62] Ross S., Cheung E., Petrakis T. G., Howell M., Kraus W. L. and Hill C. S. (2006). Smads orchestrate specific histone modifications and chromatin remodeling to activate transcription. *EMBO J.* 25, 4490-4502. 10.1038/sj.emboj.7601332 16990801 PMC1589990

[DEV184036C83] Scheiffele P. (2003). Cell-cell signaling during synapse formation in the CNS. *Annu. Rev. Neurosci.* 26, 485-508. 10.1146/annurev.neuro.26.043002.094940 12626697

[DEV184036C63] Schmidt J. E., Suzuki A., Ueno N. and Kimelman D. (1995). Localized BMP-4 mediates dorsal/ventral patterning in the early Xenopus embryo. *Dev. Biol.* 169, 37-50. 10.1006/dbio.1995.1124 7750652

[DEV184036C64] Shi Y. and Massagué J. (2003). Mechanisms of TGF-β signaling from cell membrane to the nucleus. *Cell* 113, 685-700. 10.1016/S0092-8674(03)00432-X 12809600

[DEV184036C65] Shi Y., Wang Y.-F., Jayaraman L., Yang H., Massagué J. and Pavletich N. P. (1998). Crystal structure of a Smad MH1 domain bound to DNA: insights on DNA binding in TGF-β signaling. *Cell* 94, 585-594. 10.1016/S0092-8674(00)81600-1 9741623

[DEV184036C66] Silvotti L., Cavaliere R. M., Belletti S. and Tirindelli R. (2018). *In-vivo* activation of vomeronasal neurons shows adaptive responses to pheromonal stimuli. *Sci. Rep.* 8, 8490 10.1038/s41598-018-26831-5 29855521 PMC5981476

[DEV184036C67] Soji K., Doi S., Nakashima A., Sasaki K., Doi T. and Masaki T. (2018). Deubiquitinase inhibitor PR-619 reduces Smad4 expression and suppresses renal fibrosis in mice with unilateral ureteral obstruction. *PLoS ONE* 13, e0202409 10.1371/journal.pone.0202409 30114247 PMC6095583

[DEV184036C68] Stowers L., Holy T. E., Meister M., Dulac C. and Koentges G. (2002). Loss of sex discrimination and male-male aggression in mice deficient for TRP2. *Science* 295, 1493-1500. 10.1126/science.1069259 11823606

[DEV184036C69] Strittmatter S. M., Valenzuela D., Kennedy T. E., Neer E. J. and Fishman M. C. (1990). G0 is a major growth cone protein subject to regulation by GAP-43. *Nature* 344, 836-841. 10.1038/344836a0 2158629

[DEV184036C70] Strittmatter S. M., Vartanian T. and Fishman M. C. (1992). GAP-43 as a plasticity protein in neuronal form and repair. *J. Neurobiol.* 23, 507-520. 10.1002/neu.480230506 1431834

[DEV184036C71] Südhof T. C. (2017). Synaptic neurexin complexes: a molecular code for the logic of neural circuits. *Cell* 171, 745-769. 10.1016/j.cell.2017.10.024 29100073 PMC5694349

[DEV184036C72] Tadokoro T., Gao X., Hong C. C., Hotten D. and Hogan B. L. M. (2016). BMP signaling and cellular dynamics during regeneration of airway epithelium from basal progenitors. *Development* 143, 764-773. 10.1242/dev.126656 26811382 PMC4813333

[DEV184036C73] Taroc E. Z. M., Prasad A., Lin J. M. and Forni P. E. (2017). The terminal nerve plays a prominent role in GnRH-1 neuronal migration independent from proper olfactory and vomeronasal connections to the olfactory bulbs. *Biol Open* 6, 1552-1568. 10.1242/bio.029074 28970231 PMC5665474

[DEV184036C74] Timmer J. R., Wang C. and Niswander L. (2002). BMP signaling patterns the dorsal and intermediate neural tube via regulation of homeobox and helix-loop-helix transcription factors. *Development* 129, 2459-2472. 11973277 10.1242/dev.129.10.2459

[DEV184036C75] Vassar R., Chao S. K., Sitcheran R., Nuñez J. M., Vosshall L. B. and Axel R. (1994). Topographic organization of sensory projections to the olfactory bulb. *Cell* 79, 981-991. 10.1016/0092-8674(94)90029-9 8001145

[DEV184036C76] Wakabayashi Y. and Ichikawa M. (2007). Distribution of Notch1-expressing cells and proliferating cells in mouse vomeronasal organ. *Neurosci. Lett.* 411, 217-221. 10.1016/j.neulet.2006.09.088 17123719

[DEV184036C77] Wang F., Nemes A., Mendelsohn M. and Axel R. (1998). Odorant receptors govern the formation of a precise topographic map. *Cell* 93, 47-60. 10.1016/S0092-8674(00)81145-9 9546391

[DEV184036C78] Wang X., Harris R. E., Bayston L. J. and Ashe H. L. (2008). Type IV collagens regulate BMP signalling in Drosophila. *Nature* 455, 72-77. 10.1038/nature07214 18701888

[DEV184036C79] Weiler E., McCulloch M. A. and Farbman A. I. (1999). Proliferation in the vomeronasal organ of the rat during postnatal development. *Eur. J. Neurosci.* 11, 700-711. 10.1046/j.1460-9568.1999.00476.x 10051771

[DEV184036C84] Yamagata M., Sanes J. R. and Weiner J. A. (2003). Synaptic adhesion molecules. *Curr. Opin. Cell Biol.* 15, 621-632. 10.1016/s0955-0674(03)00107-8 14519398

[DEV184036C80] Yang X., Li C., Herrera P.-L. and Deng C.-X. (2002). Generation of Smad4/Dpc4 conditional knockout mice. *Genesis* 32, 80-81. 10.1002/gene.10029 11857783

[DEV184036C81] Zhou Y., Zhou B., Pache L., Chang M., Khodabakhshi A. H., Tanaseichuk O., Benner C. and Chanda S. K. (2019). Metascape provides a biologist-oriented resource for the analysis of systems-level datasets. *Nat. Commun.* 10, 1523 10.1038/s41467-019-09234-6 30944313 PMC6447622

