# Supplementary material for: Smad4-dependent morphogenic signals control the maturation and axonal targeting of basal vomeronasal sensory neurons to the accessory olfactory bulb
Source: Development. 2020 Apr 27;147(8):dev184036. doi: 10.1242/dev.184036 (PMC7197725; doi:10.1242/dev.184036)
Supplement: Supplementary information [file develop-147-184036-s1.pdf]

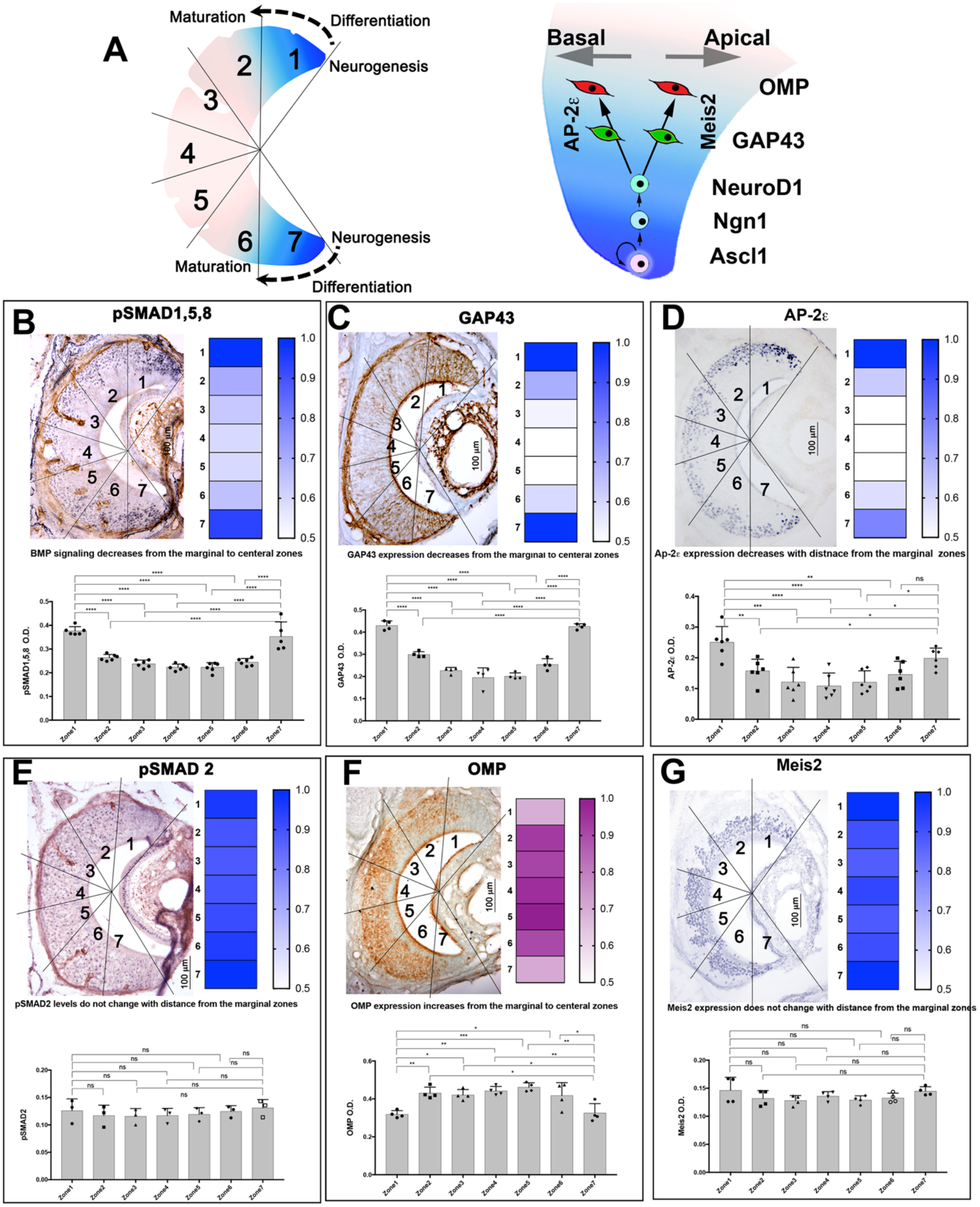

Fig. S1: Spatial correlation between the ability to transduce BMP and express AP-2ε and GAP43.

A) Cartoon illustrating neurogenesis, differentiation and maturation in the VNE. Most neurogenesis occurs at the margins followed by differentiation into apical or basal VSNs

Development • Supplementary information

which then undergo maturation. The Ascl1 neuronal progenitor cell self-renews and divides to form first Ngn1 and then NeuroD positive precursors. These then differentiate into AP-2ε positive basal VSN or Meis2 positive apical VSN. These immature neurons begin expressing GAP43. Upon reaching functional maturity, they express olfactory marker protein (OMP). B) Immunohistochemistry for pSmad 1,5,8 and its densitometric analysis along the marginal, intermediate and central zones of VNE. Graph and heat map showing cells strongly positive for pSmad1,5,8 are confined to marginal zones with immunoreactivity significantly decreasing towards the central zones. C) Immunohistochemistry for the immature marker GAP43 and its densitometric analysis along the VNE. Graph and heat map show immature neurons are largely present in the marginal zones of VNE. D) Immunohistochemistry for AP-2ε and its densitometric analysis. Graph and heat map show strong AP-2ε expressing cells in the marginal zones on VNE. E,F,G) Immunohistochemistry and densitometric analysis of pSmad2, OMP and Meis2. Graph and heat map show equal distribution of cells expressing these markers in all the zones of the VNE. OD measured in each zone following DAB staining after dividing the VNE in 7 zones, unpaired t-Test p (*<0.05), +/- SEM, (N=3 animals;3-4 sections per animal).

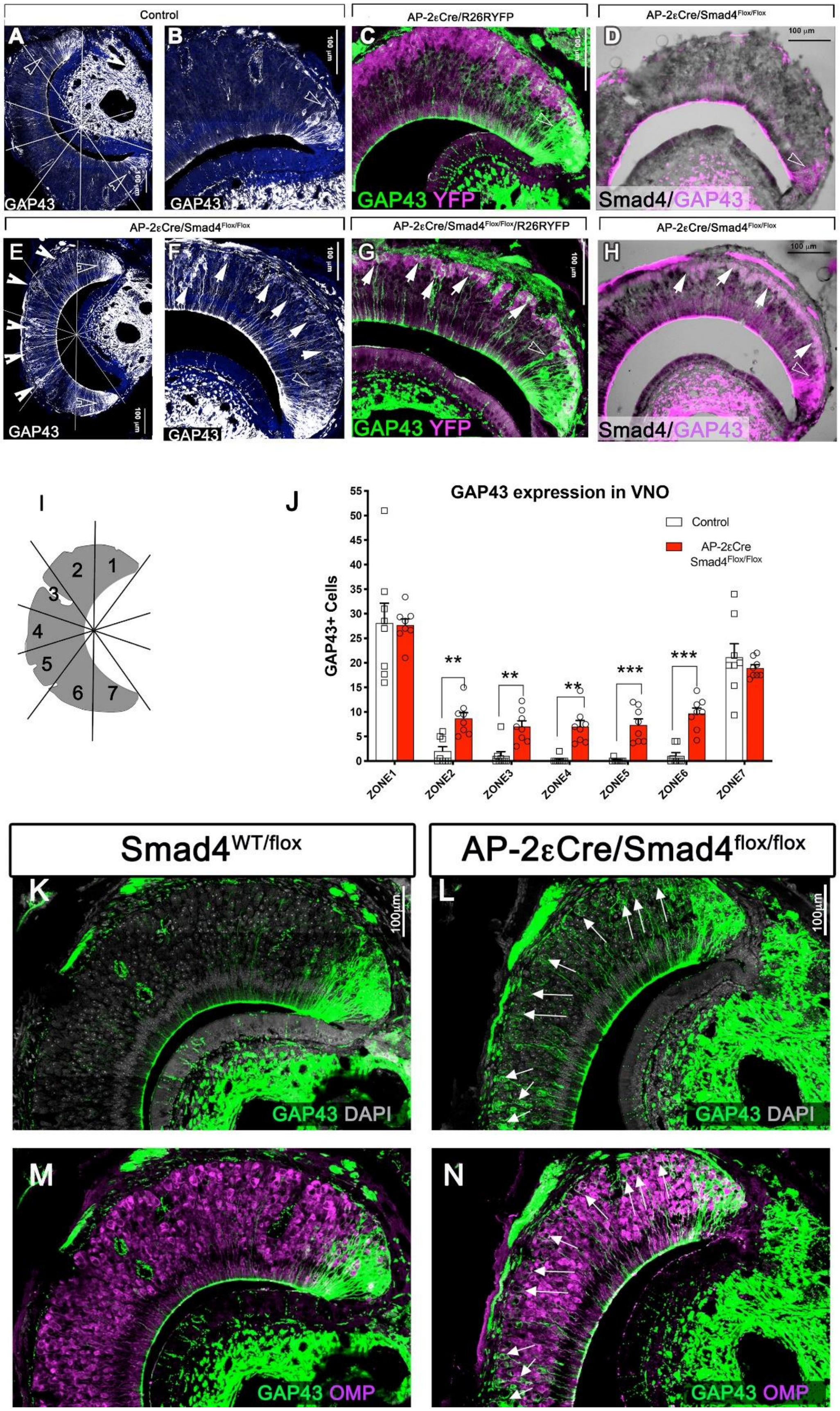

Development • Supplementary information

Fig. S2. Increased GAP43 expression after early Smad4 conditional ablation. A,E) GAP43 immunostaining in control (A) and cKO (E). White notched arrows point at GAP43 positive cells in the medial regions of VNE in the cKO (E), while in control GAP43 positive cells are mostly restricted to the marginal regions, empty notched arrows(A). B,F) magnification of A and E respectively. C,G) Immunostaining for AP-2ε driven recombination (YFP, magenta) and GAP43 (green) in control (C) and cKO (G). White arrows point to positive GAP43 positive cells in the medial regions of VNE in cKO. Empty arrows point to non-traced GAP43 positive cells, which are restricted to marginal zones (G). D,H) Immunostaining for GAP43 (magenta) and Smad4 (black) in control (D) and cKO (H). I) Cartoon illustrating how the VNE was divided in 7 zones for quantification purposes. J) Graph shows a significant increase in GAP43 positive cells in the medial regions of VNE in CKO and a comparable number of GAP43 positive cells in marginal regions in control and cKO (n=6). K,L) Immunostaining against GAP43 (green) on P60 control (K) and on P60 cKO (L). White arrows point to GAP43 positive VSNs in medial regions of VNE of the cKO. M,N) Immunostaining against GAP43 (green) and OMP (magenta) on P60 control (M) and cKO (N). White arrows point to the GAP43/OMP double positive VSNs in cKO (N).

Development • Supplementary information

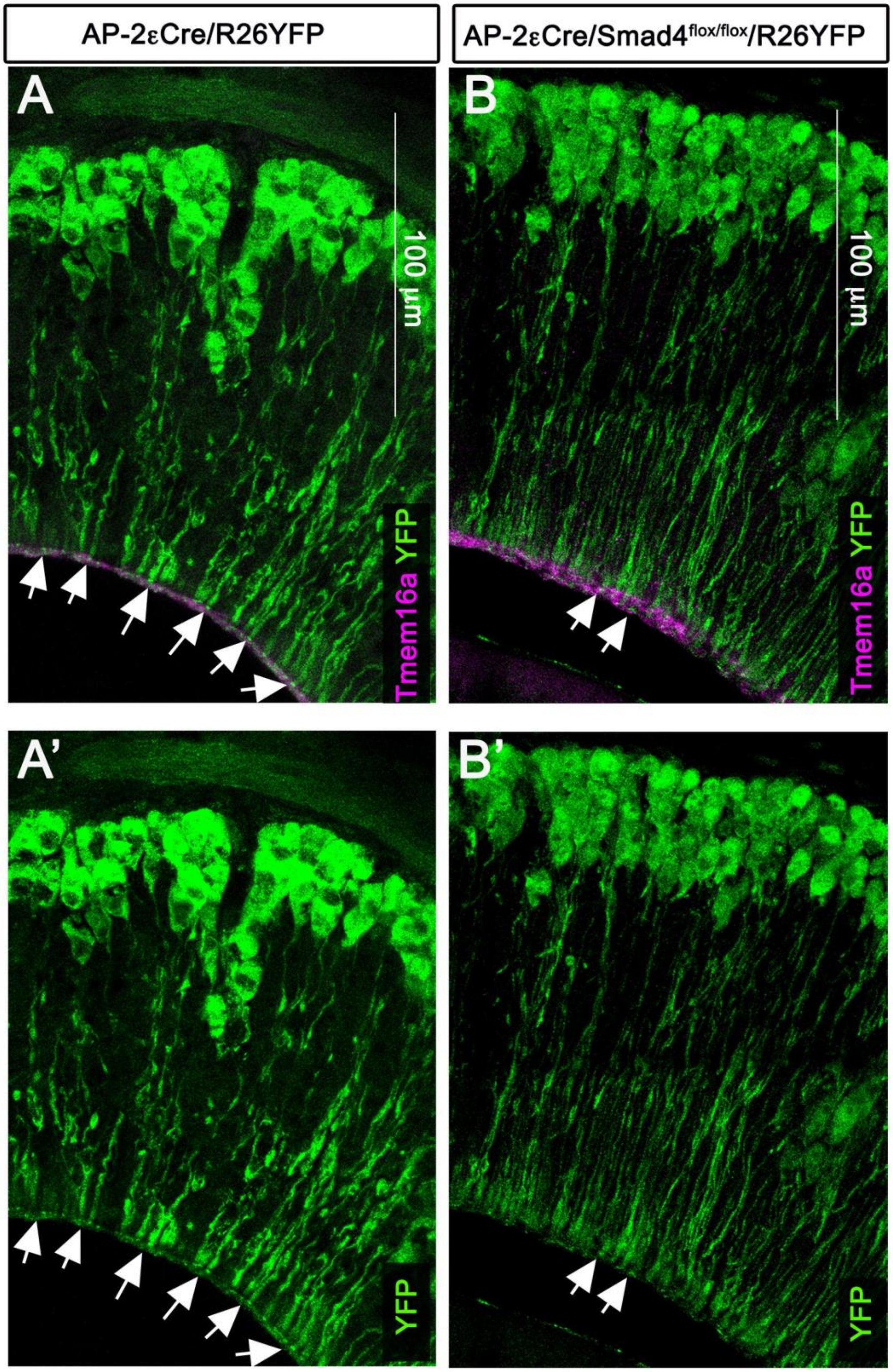

Fig S3. C) A,B) Immunostaining against Tmem16a (magenta) and YFP tracing (green) on P18 AP-2εCre/R26YFP control (A) and AP-2εCre/Smad4$^{flox/flox}$/R26YFP cKO (B). A',B') YFP tracing (green) only. White arrows point to many recognizable dendritic knobs (bright spots in the apical region) in control while few identifiable knobs were found in cKO.

Development • Supplementary information

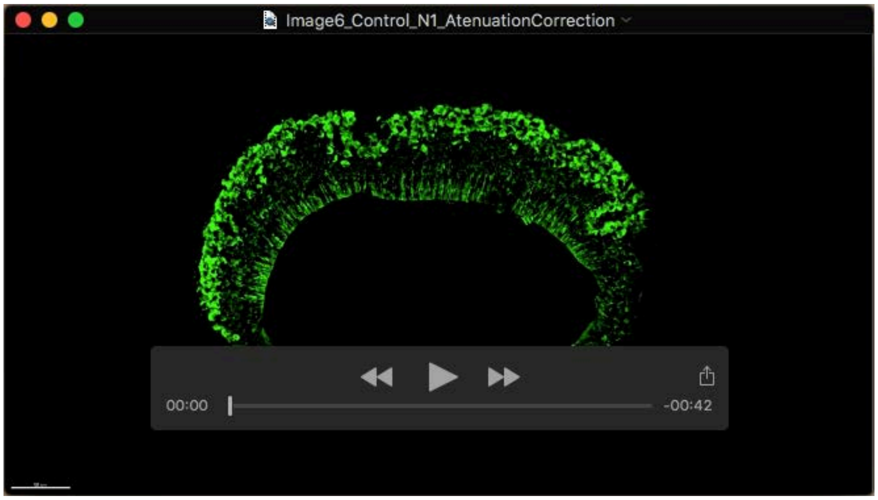

Movie 1. illustrating the method used to quantifying dendritic knobs. AP- 2εCre+/-/ R26R$^{YFP+/-}$ control. Movie shows AP-2εCre traced basal neurons (green), OMP (purple), Villin (red). Red and green objects respectively highlight apical and basal knobs based on YFP fluorescence threshold.

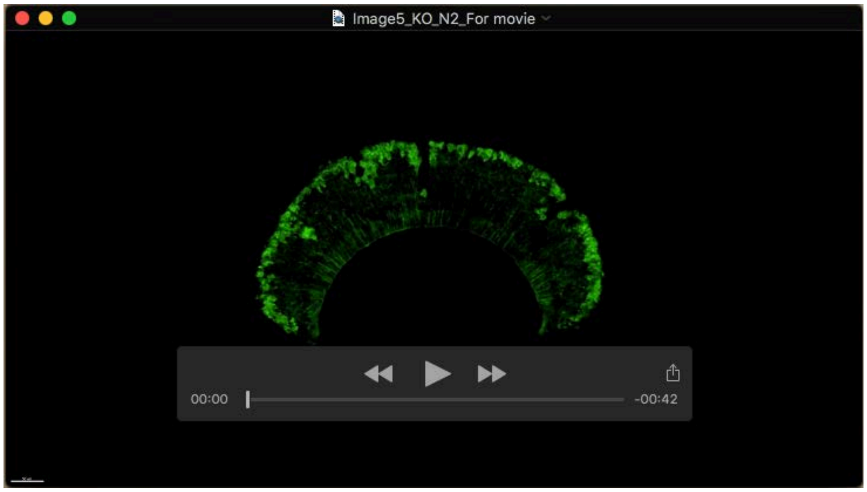

Movie 2. illustrating the method used to quantifying dendritic knobs. AP- 2εCre$^{+/-}$/ R26R$^{YFP+/-}$/Smad4$^{flox/flox}$. AP-2εCre traced basal neurons (green), OMP (purple), Villin (red). Red and green objects respectively highlight apical and basal knobs, in this sample no basal dendritic knobs had YFP fluorescence threshold comparable to controls.

Development • Supplementary information

S. Table 1. Primers used for RT-PCR			
**Oligo sequence 5'-3'**	**Oligo name**	**Tm**	**Amplification product**
CAGCCCGATCACCTCTCTTC	BMP 2_fwd	59.9	573 bp
TCCACGGCTTCTTCGTGATG	BMP2_rev	60.39	
AACCAATGAGACACCATGATTCC	BMP4_fwd	58.98	730 bp
CCTCAATGGCCAGCCCATAA	BMP4_rev	60.11	
TGGTGACTCGGGATGGACTC	BMP6_fwd	60.97	722 bp
AAAAGCCTCCGTGGGTAAGG	BMP6_rev	59.96	
GGCCTGCAAGAAACATGAGC	BMP7_fwd	60.11	170 bp
AGTGAACCAGTGTCTGGACGA	BMP7_rev	61.03	
CGCAACAACGCCATCTATGAG	TGFB1_fwd	60	295 bp
TTCCGTCTCCTTGGTTCAGC	TGFB1_rev	59.97	
TGCTCTGTGGGTACCTTGATG	TGFB2_fwd	59.72	444 bp
TAGAGGTGCCATCAATACCTGC	TGFB2_rev	59.9	
AAGCCAGCTCTTGGGTCATC	BMP3_fwd	60.03	782 bp
TTTCAAAGAACTTAGCATACTCCTC	BMP3_rev	57.09	
AAGACGCTGAACTTGTCGGA	BMP10_ fwd	59.61	152 bp
AACAGATCTTCGTTCTTGAAGC	BMP10_Rev	57	
CCCCATGCCCAAGATTGTCC	GDF10_fwd	61.04	386 bp
TACCACCAGCACGGAAGAAC	GDF10_rev	59.97	

S. Table 2. Primers used for qPCR	
**Oligo sequence 5'-3'**	**Oligo name**
TCACCGACCAGCCTCACAT	Nrxn1 Fwd
AGTTCTGAATATGGCAGCAGAGAA	Nrxn1 Rev
AGTTTGCTGAGCGAGTAGTCGAT	Tenm2 Fwd
CGGGACGGCCTATGCA	Tenm2 Rev
GAGACGCATCATCAAAGAAAACC	Unc13c Fwd
AAGTGAAGTCTAATGGCAGTGTAAAAAT	Unc13c Rev
CCCTTTCTCCCACCATATTGGT	Robo2 Fwd
GGAAGCTGATGATCTCTAATACTAGGAAAA	Robo2 Rev
ACAGACGTACCTTCCTCACCA	UBC Fwd
CCCCATCACACCCAAGAACAA	UBC Rev

S. Table 3

Downregulated in OMPCre Smad4Flox/Flox vs controls (Smad4flox/flox and OMPCre +/-)

		Term	Description	P value	LogP	Log(q-value)	InTerm_InList	Genes	Symbols
		R-MMU-217	TGF-beta receptor signaling activates SMADs	0.007285685	-2.1375296	-0.046	3/21	17126,17128	Smad2,Smad4,Tgfbr1
		R-MMU-111	Apoptotic cleavage of cellular proteins	4.8008E-06	-5.3186864	-1.359	7/36	12370,16905	Casp8,Lmna,Ocln,Plec,Satb1,Stk26,Gsn,Casp9,Mapk8
		R-MMU-885	Clathrin-mediated endocytosis	0.004680007	-2.3297535	-0.126	8/135	13855,14269	Epn2,Fnbp1,Ldlr,Pip5k1c,Pacsin2,Arrb1,D130043K22Rik,Arfgap1
		mmu04110	Cell cycle	2.83801E-05	-4.5469863	-0.888	11/124	12236,12448	Bub1b,Ccne2,Mad1l1,Smad2,Smad4,Mcm3,Mcm2,Mcm5,Cdc6,Skp2,Cdc20,Esco2,Mapk8,Poli,Chaf1a,Tspyl2,Fancm
		GO:0051056	regulation of small GTPase mediated signal transduction	4.5913E-05	-4.3380645	-0.869	16/259	11517,11857	Adcyap1r1,Arhgdib,Eps8,Cyth1,Vav1,Icmt,Stambp,Ralgps2,Arrb1,Mfn2,Ripor2,P2ry10b,Cdc42bpa,Mapkap1,Arfgap1,Arhgap25,Plaat1,Setdb1,Rab15
		R-MMU-287	FCERI mediated MAPK activation	5.18957E-05	-4.2848686	-0.869	5/22	17444,22324	Grap2,Vav1,Map2k7,Mapk10,Mapk8,Pdpk1,Fbxw11,Ciita,Casp8,Casp9,Ldlr,Tlr11,Csf2rb,Daxx,Lmna,Sptan1,Mapk7,Itgb2,Arhgdib,Ntf3,Tap1,Skp2,Gtf2ird1,Ifih1,Taf5l,Mavs
		GO:0000302	response to reactive oxygen species	0.005748264	-2.2404633	-0.090	10/201	18391,19229	Sigmar1,Ptk2b,Sirpa,Sphk1,Mapk7,Mapk8,Stk26,Plekha1,Prkaa1,Hk3
		GO:0010638	positive regulation of organelle organization	0.000241309	-3.6174266	-0.635	25/595	11733,13163	Ank1,Daxx,Lmna,Smad4,Ntf3,Ptk2b,Sphk1,Tgfbr1,Map2k7,Mapk8,Sept9,Clip1,Nek7,Cnot2,C2cd5,Setdb1,Prkaa1,Arrb1,Mfn2,Arid5a,Hmbox1,Gsn,5330417C22Rik,Sept7,Nav3,Bub1b,Mad1l1,Mcm2,Top2a,Cdc6,Naa10,Kdm4c
		GO:0030029	actin filament-based process	0.000598629	-3.2228422	-0.439	28/743	11519,11857	Add2,Arhgdib,Eps8,Kcnj8,Kit,Smad4,Myo7a,Ntf3,Ptk2b,Sirpa,Sptan1,Tgfbr1,Fmnl3,Pacsin2,Setd3,Arrb1,Myh7,Shroom4,Wipf1,Cdc42bpa,Mapkap1,Gsn,Fhdc1,Rap1gds1,Arhgap25,Scn3b,Gas2l3,Wipf3
		R-MMU-125	PIP3 activates AKT signaling	0.009531022	-2.0208605	-0.024	9/184	12371,16590	Casp9,Kit,Pdpk1,Pip5k1c,Rnf2,Vav1,Ppp2r5c,Mapkap1,Npcd

Upregulated in OMPCre Smad4Flox/Flox vs controls (Smad4flox/flox and OMPCre +/-)

GroupID	Category	Term	Description	P value	LogP	Log(q-value)	InTerm_InList	Genes	Symbols
		GO:0006904	vesicle docking involved in exocytosis	0.000669419	-3.1743018	-0.118	3/44	17967,20910	Ncam1,Stxbp1,Unc13c
		GO:0030534	adult behavior	8.32572E-06	-5.079578	-1.120	7/187	12288,12919	Cacna1c,Crhbp,Slc1a2,Homer2,Rnf180,Zmpste24,Sdk1,Ncam1,Adcyap1,Atp8a1,Nhlh2,Foxo6
		GO:0019722	calcium-mediated signaling	0.00016476	-3.7831474	-0.336	6/209	11938,17967	Atp2a2,Ncam1,Tenm2,Homer2,Camta1,Zmpste24,Adcyap1
		GO:0061025	membrane fusion	0.000177061	-3.751877	-0.336	5/134	20362,20910	Sept8,Stxbp1,Gosr2,Mymk,Trarg1,Atp2a2,Atp8a1,Epb41l3,Zmpste24,Unc13c
		GO:0051968	positive regulation of synaptic transmission, glutamatergic	0.000433612	-3.362899	-0.118	3/38	11516,20910	Adcyap1,Stxbp1,Iqsec2,Unc13c
		GO:0043269	regulation of ion transport	0.002099797	-2.6778228	0.000	9/731	11516,11980	Adcyap1,Atp8a1,Cacna1c,Crhbp,Stxbp1,Homer2,Tesc,Syt13,Zmpste24
		GO:1990778	protein localization to cell periphery	0.002196785	-2.6582124	0.000	6/343	13823,20910	Epb41l3,Stxbp1,Tesc,Ldlrap1,Trarg1,Iqsec2,Nup54
		GO:0070509	calcium ion import	0.00460171	-2.3370807	0.000	3/86	11938,12288	Atp2a2,Cacna1c,Zmpste24,Crhbp,Ncam1,Mymk,Tnfrsf21
		GO:0006486	protein glycosylation	0.007078303	-2.1500708	0.000	4/195	14538,76483	Gcnt2,Lmf1,Galnt16,Fktn
		GO:0070372	regulation of ERK1 and ERK2 cascade	0.007310688	-2.1360417	0.000	5/311	11516,14538	Adcyap1,Gcnt2,Camk2n1,Akap12,Arrb1

Downregulated in AP-2eCre Smad4Flox/Flox vs Controls (Smad4flox/flox and AP-2eCre +/-)

GroupID	Category	Term	Description	P value	LogP	Log(q-value)	InTerm_InList	Genes	Symbols
		GO:0035082	axoneme assembly	1.5095E-06	-5.8211681	-1.938	13/68	19888,21821	Rp1,Ift88,Ccdc39,Dnaic1,Spag17,Ccdc151,Ak7,Dnah5,Zmynd10,Cfap44,Dnah7a,Cfap43,Dnah7c,Invs,Dnah10,Bbs4,Ropn1l,Cfap54,Atg5,Stil,Sept9,Fam161a,Celsr3,Ccdc88a,Dync2h1,Nudcd3,Ubxn10,Iqub,Gsn,Cdc14a,Abcc4,Ablim3,Tmem67,Clasp1,Prc1,Capn3,Grb7,Mef2c,Pdcd6ip,Pip4k2a,Rdx,Six4,Ldb3,Mapk8,Srpx,Chmp4c,Map1lc3a,Stx17,Washc1,Mypn,Stx18,Cnot2,Tubgcp2,Vmp1,Mfn2,Shank2,Pacs2,Rab7b,Ccdc136,Nsfl1c,Bin2,Cttn,Lpar1,Pmp22,Sh2b1,Espn,Ttyh1,Eps8l1,Ap3s1,Cnp,Diaph1,Klc2,Ktn1,Met,Camsap3,Dclk2,Ints13,Clasp2,Skap1,Cdc42bpa,Madd,Dnah9,Nav3,Casc1,Saxo2
		GO:0006888	ER to Golgi vesicle-mediated transport	0.007544669	-2.1223598	-0.184	11/123	12068,20334	Bet1,Sec23a,Preb,Tmed2,Stx17,Rab43,Lman1,Stx18,Tmed5,Sec16b,Mia2
		GO:0042573	retinoic acid metabolic process	0.000176642	-3.7529053	-0.746	6/23	11522,11529	Adh1,Adh7,Aldh1a1,Rdh16,Akr1c18,Dhrs9,Acss1,Dab2,Kcnma1,Pecr,Napepld,Nus1
		GO:0072659	protein localization to plasma membrane	0.000183044	-3.7374442	-0.746	24/278	11651,12988	Akt1,Csk,Dab2,Itga3,Rab3d,Rdx,Sec23a,Stx3,Pacsin1,Pgrmc1,Pkp2,Rab13,Washc1,Nkd2,Grip1,Ankrd9,Clasp2,Skap1,Sytl2,Camk2d,Pacs2,Scn3b,Rab26,Pram1,Apoe,Cacna1a,Capn3,Folr1,Itgb2,Exoc4,Snap25,Lgi1,Ssr3,Tram1,Pex26,Tm9sf3,Gsn,Frmpd1
		GO:0034330	cell junction organization	0.000302464	-3.5193267	-0.690	22/253	12340,12988	Capza1,Csk,Cttn,Ocln,Pdcd6ip,Plec,Pmp22,Ptprk,Rdx,Abcc8,Actn4,Pkp2,Rab13,Camsap3,Marveld3,Whrn,Vmp1,Clasp2,Clasp1,Pkp4,Plekha7,Grhl2
		GO:0010498	proteasomal protein catabolic process	0.000364713	-3.4380484	-0.690	33/456	11651,11816	Akt1,Apoe,Arntl,Btrc,Dab2,Pmp22,Siah1b,Psmd2,Amfr,Mapk8,Ppp2r5c,Dnajb9,Fbxl3,Erlec1,Ufl1,Stt3b,Rmnd5a,Derl3,Ankib1,Osbpl7,Usp19,Nkd2,Rhbdd1,Trim2,Kat5,Cul4a,Edem2,Mta1,Ube4a,Dlgap1,Psmf1,Tmem67,Nsfl1c,Hipk2,Pdcd6ip,Tgfb1i1,Cnot4,Chmp4c,Trim32,Cacul1,Usp43,Otud7b,Zranb1,Dnajc3,Rdx,Banp,Pacsin3,Rab26,Ptpn3
		GO:0016050	vesicle organization	0.000396505	-3.4017516	-0.677	22/258	12068,14339	Bet1,Aktip,Pdcd6ip,Ptbp1,Rab5c,Sec23a,Snap25,Stx3,Syt3,Preb,Srpx,Stx17,Washc1,Nkd2,Vps16,Sec16b,Mta1,Vps8,Rab7b,Stx16,Ccdc136,Myo18a,Akt1,Apoe,Bcl2l11,Cd36,Cnp,Itga3,Itgb2,Mbp,Plscr2,Pmp22,Psap,Sirpa,Pacsin1,Pacsin2,Sytl4,Chmp4c,Taz,Apool,Tlcd1,Stx18,Alkbh4,Grip1,Immt,Pacsin3,Ccdc88a,Mfn2,Gsn,Atp10d,Lpcat2,Nsfl1c,Bin2,Miga1
		GO:0030029	actin filament-based process	0.000523344	-3.2812127	-0.677	47/743	11519,12288	Add2,Cacna1c,Capn3,Capza1,Cttn,Diaph1,Lpar1,Mef2c,Met,Pak3,Pdcd6ip,Pmp22,Sirpa,Rdx,Six4,Fmnl3,Dnajb6,Pacsin1,Pacsin2,Ldb3,Cttnbp2,Nox4,Rufy3,Espn,Actn4,Pkp2,Rab13,Washc1,Mypn,Trim32,Alkbh4,Rhoh,Clasp2,Clasp1,Bbs4,Camk2d,Ccdc88a,Cdc42bpa,Gsn,Scn3b,Atp2c1,Myrip,Ablim3,Gpd1l,Frmpd4,Myo18a,Arhgap40,Apoe,Cd36,Krt14,Rp1,Ift88,Mapk8,Camsap3,Tubgcp2,Vipas39,Nav3
		GO:0030520	intracellular estrogen receptor signaling pathway	0.008641718	-2.0633999	-0.147	6/47	18600,22061	Padi2,Trp63,Uba5,Ufl1,Ufm1,Cnot2
		GO:0098656	anion transmembrane transport	0.000560231	-3.2516328	-0.677	17/180	11651,11988	Akt1,Slc7a2,Cftr,Folr1,Gabrb3,Slc12a2,Slc1a1,Slc1a5,Slc23a1,Slc7a7,Slc25a22,Ano1,Slc5a8,Slc35c2,Slc36a4,Slc16a12,Slc7a6,Apoe,Cacna1a,Cd36,Clca3a1,Psap,Ttyh1,Repin1,Pla2g12a,Slc52a3,Slco1a5,Clic6,Gabrp,Slc4a7,Ahcyl1,Atp10d,Tmem30b,Abcc4,Pla2g4f

Upregulated in AP-2eCre Smad4Flox/Flox vs Controls (Smad4flox/flox and AP-2eCre +/-)

GroupID	Category	Term	Description	P value	LogP	Log(q-value)	InTerm_InList	Genes	Symbols
		GO:0001944	vasculature development	0.000485684	-3.3136458	-0.686	14/751	11350,11481	Abl1,Acvr2b,Atf2,Col18a1,Epor,Hdac5,Igf2,Itgb3,Jun,Nrxn1,Sox4,Mcam,Unc5b,Robo2
		GO:0010770	positive regulation of cell morphogenesis involved in differentiation	3.97554E-06	-5.4006043	-1.532	9/184	11350,13829	Abl1,Dmtn,Itgb3,Map6,Ntn1,Pak1,Caprin1,Trak1,Robo2,Cttn,Epor,Ezh2,Itsn1,Map4,Nrxn1,Map4k4,Entr1,Abi1,Bcl2l1,Dpysl5,Unc5b,Myo16,Col18a1,Parvb,Lhfpl5,Spag9,Slc9b2,Ppp1r9b,Gnai2,Gas2,Jun
		GO:0030036	actin cytoskeleton organization	8.42226E-06	-5.0745712	-1.465	16/661	11308,11350	Abi1,Abl1,Cnn2,Cttn,Dmtn,Fhl3,Itgb3,Pak1,Pstpip1,Tln1,Inf2,Cnn3,Bcr,Parvb,Ppp1r9b,Sh3pxd2b,Map4k4,Itsn1,Gcnt2,Itga11,Map4,Nrxn1,Ntn1,Entr1,Anxa5,Ezh2,Grin2c,Jun,Dusp10,Cdkn2c,Rptor,Usp47,Map6,Bmerb1,Bicd2
		GO:0007229	integrin-mediated signaling pathway	2.85755E-05	-4.5440056	-1.193	6/89	11350,13829	Abl1,Dmtn,Itgb3,Tln1,Fyb2,Itga11,Jun,Pak1,Parvb,Anxa5,Igf2
		GO:0023061	signal release	4.64461E-05	-4.3330504	-1.152	13/522	11481,11747	Acvr2b,Anxa5,Bcl2l1,Dio2,Itsn1,Nrxn1,Pak1,Sox4,Map4k4,Pdzd11,Rptor,Slc9b2,Unc13c,Igf2,Stat5b,Srd5a1,Cnn2,Dmtn,Gnai2,Itgb3,Kcnab1,Sfrp4,Bcr,Abl1
		GO:0008637	apoptotic mitochondrial changes	9.88211E-05	-4.0051503	-1.067	6/111	11909,12048	Atf2,Bcl2l1,Jun,Plaur,Mllt11,Ndufs1,Cttn,Inf2,Abl1,Acvr2b,Col18a1,Ezh2,Pthlh,Sfrp4,Sox4,Stat5b,Lhfpl5,Anxa5,Unc5b,Trak1,Gnai2,Usp47
		GO:0045859	regulation of protein kinase activity	0.000137017	-3.8632255	-0.979	14/663	11350,11481	Abl1,Acvr2b,Cdkn2c,Ezh2,Itgb3,Nrxn1,Dusp8,Pak1,Map4k4,Dusp10,Spag9,Rptor,Magi3,Ccnjl,Igf2,Sfrp4,Atf2,Jun
		GO:0071277	cellular response to calcium ion	0.000162421	-3.7893585	-0.944	5/77	13829,16476	Dmtn,Jun,Nrxn1,Syt13,Fus,Anxa5,Abl1,Epor,Ezh2
		GO:0006887	exocytosis	0.000217787	-3.661969	-0.919	10/377	12048,13829	Bcl2l1,Dmtn,Gnai2,Itsn1,Nrxn1,Pak1,Prss12,Syt13,Bcr,Unc13c,Grin2c,Rabep1,Pdzd11,Ppp1r9b,Fyb2,Sh3pxd2b,Epor,Khsrp,Slc29a1
		GO:0045785	positive regulation of cell adhesion	0.000439636	-3.3569067	-0.719	10/412	11350,13829	Abl1,Dmtn,Gcnt2,Igf2,Itgb3,Kifap3,Stat5b,Map4k4,Dusp10,Vsir,Nrxn1,Ntn1,Mcam,Robo2,Otop1,Bcr

Development • Supplementary information
