# Supplementary material for: Smad4-dependent morphogenic signals control the maturation and axonal targeting of basal vomeronasal sensory neurons to the accessory olfactory bulb
Source: Development. 2020 Apr 27;147(8):dev184036. doi: 10.1242/dev.184036 (PMC7197725; doi:10.1242/dev.184036)
Supplement: Reviewer comments [file develop-147-184036_review_history.pdf]

Smad4-dependent morphogenic signals control the maturation and axonal targeting of basal vomeronasal sensory neurons to the accessory olfactory bulb

Ankana S. Naik, Jennifer M. Lin, Ed Zandro M. Taroc, Raghu R. Katreddi, Jesus A. Frias, Alex A. Lemus, Morgan A. Sammons and Paolo E. Forni
DOI: 10.1242/dev.184036

Editor: Liz Robertson

Review timeline

Original submission:	20 August 2019
Editorial decision:	16 October 2019
Rebuttal received:	21 October 2019
Editorial decision:	20 November 2019
First revision received:	24 January 2020
Editorial decision:	26 February 2020
Second revision received:	29 February 2020
Accepted:	10 March 2020

Original submission

First decision letter

MS ID#: DEVELOP/2019/184036
MS TITLE: Smad4 signaling establishes the somatosensory map of basal vomeronasal sensory neurons
AUTHORS: Ankana Naik, Jennifer M. Lin, Ed Zandro M. Taroc, Raghu Katreddi, Jesus A. Frias, Morgan A. Sammons, and Paolo E. Forni

Dear Dr. FORNI,

I have now received all the referees' reports on the above manuscript, and have reached a decision. I am sorry to say that the outcome is not a positive one. The referees' comments are appended below, or you can access them online: please go to Development's submission site and click on the 'Manuscripts with Decisions' queue in the Author Area.

As you will see, the referees raise some significant concerns about your paper, and are not strongly in favour of publication in Development. Having looked at the manuscript myself, I agree with their views, and I must therefore, reject your paper.

I do realise this is disappointing news, but Development receives many more papers than we can publish, and we can only accept manuscripts that receive strong support from referees.

I do hope you find the comments of the referees helpful, and that this decision will not dissuade you from considering Development for publication of your future work. Many thanks for sending your manuscript to Development.

Reviewer 1 Advance Summary and Potential Significance to Field:

Understanding how various signaling molecules regulate the specific outgrown and synaptic connections of axons to their targets is a major question in developmental neuroscience. One of

© 2020. Published by The Company of Biologists under the terms of the Creative Commons Attribution License (https://creativecommons.org/licenses/by/4.0/).

the best studied systems in mammals is the olfactory sensory neuron connectivity to glomeruli targets in the olfactory bulb, which allows perception of odorants in space. Many animals, including humans, also have an accessory system for detecting pheromones, known as the vomeronasal organ (VNO). Although this system plays a key role in multiple behaviors, it is much less studied. An RNAseq study found that multiple components of the TFG-beta family are highly expressed in the mouse postnatal VNO. To follow this clue, the authors used novel Cre-lines of mice to investigate whether TGF-beta signaling impacts the specificity of vomeronasal sensory neuron projections to the accessory olfactory bulb. This is an important question, the premise for the study is valid.

Reviewer 1 Comments for the Author:

For the most part, the experiments are well thought out and the data are convincingly presented. The validation of the basal cell conditional SMAD4 KO is well done and convincing. The immunostaining showing that these mice have nearly normal numbers of VSNs at P15 is of high quality. By looking at P60 mice when connections to the AOB should have formed, the authors found that basal VSNs selectively do not mature, respond to pheromones or form an organized glomerular layer in the AOB. Following up with physiological stimulation by pheromones takes the descriptive morphological data to a functional level. Thus, the data presenting in this manuscript are novel and make an important contribution to the field. However, although this manuscript is very data-rich, in my opinion it will be hard to follow for the non-specialist. The manuscript needs a complete rethinking to focus on the importance of the findings.
Suggestions for improving clarity:

1) Neither the abstract nor the introduction communicate the importance of the findings. They simply say SMAD4 is necessary, but what makes this novel and how does it address specificity? The reader needs to be exposed to the significance of the work early in the manuscript.
2) Localization of active BMP signaling section. Fig 1A&B: The RT-PCR and RNAseq levels of the ligand transcripts often do not match – this should be discussed. The rationale for the various analyses needs to be more precise. For example, analyzing the Arx-1 null mice is not clearly explained; it may be obvious to a neuroscientist but perhaps not to a more generalized readership.
3) Overall, the rationale for the observations reported in each “results” section needs to be clearer, and the conclusions should include the significance of these findings. The authors need to tell me what makes these findings important.
4) Same comment for the discussion section.

Minor points:
1) I am not sure that the DevBio community would understand that male urine contains pheromones – the rationale for this stimulus should be briefly explained.
2) “data” are plural – this needs to be fixed here and there in the manuscript.

Reviewer 2 Advance Summary and Potential Significance to Field:
This paper provides evidence of the consequences of targeted loss of function of Smad4, a non-specific mediator of TGF-beta signaling via complexing with Smad 1/5/8 in two classes of cells in the vomeronasal organ. The core data describes the modest changes that result from Smad4 inactivation in undifferentiated cranial epithelial cells, and compares these changes with slightly less modest changes, mostly degenerative, resulting from inactivation in mature vomeronasal sensory neurons. There is little in the data to support claims of "Bmp"gradients, and the role of Smad4 in establishing a "somatosensory map". Indeed, if this refers to topological organization of sensory neurons that express different classes of pheromone receptor proteins, then that issue is not really addressed at all by the data presented. Thus, the paper is basically a long descriptive exercise reporting inactivating a fairly ubiquitous TGF-beta signaling intermediary in an interesting population of chemosensory neurons. The modest nature of the changes due to loss of function and several other substantive issues of data presentation and interpretation—particularly failure to define clearly the relationship between experimental questions and data presented—make the level of advance offered by this paper relatively modest. It is unlikely to have a major impact on current developmental analysis of TGF-beta signaling and the role of Smad signaling proteins, nor on investigation of the vomeronasal epithelium as a continually regenerating neuroepithelium.

© 2020. Published by The Company of Biologists under the terms of the Creative Commons Attribution License (https://creativecommons.org/licenses/by/4.0/).

Reviewer 2 Comments for the Author:
The individual histological images are of high quality; nevertheless, the images, and the quantification presented rarely supports the interpretations that emerge: for example, it is not clear how one can go from the localization of vascular cells with PECAM, the unsurprising decline of pSMAD 1/5/8 cells based upon distance from the basal lamina, and some sort of "gradient" of Smad, or Bmp signaling.

Similarly, it is not clear how this data in Figure 1 really addresses Smad4 signaling function at all, since it is not made clear whether Smad4 has any specific functional role in the transduction of TGF-beta signals in vomeronasal progenitors or neurons, aside from its localization, along with other Smads. The apparently ubiquitous expression of Smad4 reinforces uncertainty that there is any specific role, and that the conditional inactivation will be informative.

The comparison of the AP2:Cre versus OMP:Cre results is not well formulated. The authors suggest a tenuous relationship, but there are other more straightforward explanations: two different but related cell types, at different stages of their life cycle, have modest changes due to selective loss of Smad4 function.

The changes in glomerular organization, while interesting, do not really address "a somatosensory map" or really any issue of the potential topological organization of functional or molecular subclasses of vomeronasal sensory neurons that project to the accessory olfactory bulb.

Author rebuttal letter

Dear Editors,

After two months of waiting we were quite disappointed to learn that our manuscript entitled "Smad4 signaling establishes the somatosensory map of basal vomeronasal sensory neurons" has been rejected. In particular, after reading of the comments provided by reviewer 2, we would like to express our serious concern that this reviewer did not carefully read our manuscript as several of their statements are incorrect.

For example, they state "*The core data describes the modest changes that result from Smad4 inactivation in undifferentiated cranial epithelial cells, and compares these changes with slightly less modest changes, mostly degenerative, resulting from inactivation in mature vomeronasal sensory neurons.*"

In contrast to their statement we specifically ablate Smad4 expression in maturing differentiated basal vomeronasal sensory neurons (VSNs), using an AP-2ε Cre line that we have previously characterized and published (Lin et al., 2018). Moreover, after AP-2ε Cre mediated Smad4 conditional ablation in the basal vomeronasal neurons we observed not a "*modest*" but very severe phenotype with an approximately 50% decrease in the number of basal VSNs. The magnitude of the cell loss that we observed in adult AP-2ε Cre Smad4 cKO is as severe as the one reported for $G\alpha o$ and $G\gamma 8$ mutants (Chamero et al., 2011; Montani et al., 2013) and the connectivity to the brain is as disrupted as for the Kirrel2/Kirrel3 double mutants (Prince et al., 2013). The fact that we did not detect any obvious phenotype at two weeks after birth (Fig.3) but in adult mice suggests an important temporal requirement for SMAD4 and BMP signaling in the development of a functional vomeronasal system.

"*…..and compares these changes with slightly less modest changes, mostly degenerative, resulting from inactivation in mature vomeronasal sensory neurons.*"

Unlike what stated by the reviewer, ablation of Smad4 in mature VSNs (Smad4;OMP-Cre mice) does not lead to "*slightly less modest changes, mostly degenerative*", on the contrary these mice showed no change in the number of VSNs, no defect is odor detection but altered the connectivity, aberrant glomeruli, of the basal VSNs in the accessory olfactory bulb. These two models support evidence that Smad4 signaling is necessary for the correct circuit formation of basal vomeronasal sensory neurons.

© 2020. Published by The Company of Biologists under the terms of the Creative Commons Attribution License (https://creativecommons.org/licenses/by/4.0/).

“The modest nature of the changes due to loss of function and several other substantive issues of data presentation and interpretation—particularly failure to define clearly the relationship between experimental questions and data presented—make the level of advance offered by this paper relatively modest.”

We would be happy to edit the paper taking in account this comment. However, contrary to what stated by the reviewer we believe that the effects observed after Smad4 ablation are far from modest.

“It is unlikely to have a major impact on current developmental analysis of TGF-beta signaling and the role of Smad signaling proteins, nor on investigation of the vomeronasal epithelium as a continually regenerating neuroepithelium.”

If and what roles TGFbeta signaling play in neuroepithelia is an ongoing relevant question in the field. The fact that the VNO is a highly regenerative organ does not change the message or anything in our conclusions as we ablated Smad4 in postmitotic cells and followed the impact on differentiated neurons.
We showed that by silencing Smad4 mediated gene transcription VSNs at different maturation stages has dramatic effects on functionality of the neurons, homeostasis and connectivity to the brain. Our work could change the perspective on how people think about BMPs in neuroepithelia. In fact, differently from what shown in neuro muscular junctions (Berke et al., 2013) and trigeminal nerve (Hodge et al., 2007), where BMP is retroactively transported to the neurons we show that the vomeronasal epithelium is a source if BMPs and we show that BMP signaling gradients within the epithelium might be dictated by the relative position of neurons with respect to the vasculature and the basal lamina.
Moreover, we provide, for the first time, evidence that suggest a fundamental role for morphogenic signaling in determining the organization of vomeronasal glomeruli. Our data RNA-seq show that after loss of canonical BMP signaling there are dramatic changes in gene expression and up regulation of a number of surface molecules.
Our work points to an active role for BMP signaling in tuning gene expression levels in neurons and that this could be a key component in defining glomeruli organization.
We believe that the message is new and relevant.

“The individual histological images are of high quality; nevertheless, the images, and the quantification presented rarely supports the interpretations that emerge: for example, it is not clear how one can go from the localization of vascular cells with PECAM, the unsurprising decline of pSMAD 1/5/8 cells based upon distance from the basal lamina, and some sort of "gradient" of Smad, or Bmp signaling.”

We showed that Collagen IV is expressed by the basal lamina and by the PECAM positive vasculature. Collagen IV has been shown to pay a role in creating BMP signaling gradients by facilitating BMP-BMPR interactions (Wang et al., 2008). We quantified a decrease in intracellular pSMAD1,5,8 immunoreactivity that correlates with the distance from sources of collagen IV.

“….the unsurprising decline of pSMAD 1/5/8 cells based upon distance from the basal lamina, and some sort of "gradient" of Smad, or Bmp signaling.”

As far as we know this is the first time that data supporting a spatial relationship between sources of Collagen IV and intensity of Smad 1,5,8, phosphorylation signaling had been reported in mammals. We would like to stress that contrary of what stated by the reviewer we neither showed BMP gradients nor stated that there are Smad gradients. We reported the existence of an active BMP signaling gradient revealed by different levels of phosphorylation of SMAD1,5,8 in the VNO. Moreover, as described in the text, this observation was also important to show that while Smad2 (TGF signaling) is phosphorylated in both apical and basal neurons BMP singling (pSMAD1,5,8) is activated only in the basal neurons (Fig1 J-M). This was further demonstrated by pairing pSMAD1,5,8 with Ap2eCre genetic lineage tracing (Fig1 I).

“Similarly, it is not clear how this data in Figure 1 really addresses Smad4 signaling function at all, since it is not made clear whether Smad4 has any specific functional role in the transduction

© 2020. Published by The Company of Biologists under the terms of the Creative Commons Attribution License (https://creativecommons.org/licenses/by/4.0/).

of TGF- beta signals in vomeronasal progenitors or neurons, aside from its localization, along with other Smads. The apparently ubiquitous expression of Smad4 reinforces uncertainty that there is any specific role, and that the conditional inactivation will be informative."

We find a big conceptual gap in this comment.
Smad4 is a necessary molecular component of the BMP/TGF beta signaling machinery. Without Smad4 there is no canonical BMP/TGF dependent gene transcription.
We are well aware that Smad4 is broadly expressed. We never stated that Smad4 is selectively expressed in the VNO. We exploited Smad4 as genetic target to silence the canonical BMP intracellular signaling. Smad4 conditional ablation has been extensively used an accepted by the community as a valid approach to test roles of BMP/TGF signaling in defined spatial/temporal conditions (Colak et al., 2008; Retting et al., 2009; da Silva et al., 2011; Tong and Kwan, 2013).
Our results extensively confirm that Smad4 is needed in the VNO at multiple maturation stages. Moreover, the fact that we observed Smad4 dependent phenotypes limited to the basal vomeronasal population, where BMP signaling is active, supports the validity of our approach directed to silencing BMP signaling. In addition to this, as part of the submitted study we deposited a large amount of data obtained using RNA-seq from all the Smad4 conditional models that demonstrate important roles for BMP-Samd4 dependent signaling in controlling gene expression in the vomeronasal organ.

"*The comparison of the AP2:Cre versus OMP:Cre results is not well formulated. The authors suggest a tenuous relationship, but there are other more straightforward explanations: two different but related cell types, at different stages of their life cycle, have modest changes due to selective loss of Smad4 function.*"

We silenced BMP signaling in maturing basal VSNs (Ap-2εCre) and in mature apical and basal VSNs (OMPCre). We do not see effects of Samd4 ablation on "*two different but related cell types*", we only see effects <u>on basal VSNs</u> which are the ones transducing BMP signaling.
Ap-2εCre and OMPCre mediated Samd4 ablation give aberrant and disorganized glomeruli as convergent phenotype. We believe that our approach is compelling.

"*The changes in glomerular organization, while interesting, do not really address "a somatosensory map" or really any issue of the potential topological organization of functional or molecular subclasses of vomeronasal sensory neurons that project to the accessory olfactory bulb.*"

We believe that the dramatic phenotypes and the changes in expression of genes previously implicated in circuit assembly e.g. Neurexin1 (Sudhof, 2017) and Tenm-2 (Hong et al., 2012) point to a potential role for BMP signaling in tuning gene expression in neuronal subclasses.
We would be happy to rephrase changes in somatosensory map with changes in changes in glomerular organization.

For the specific reasons explained above we would like to appeal and ask to the editors to reconsider their final decision. We would like to resubmit a revised version of the paper; we would also ask to exclude reviewer 2 from any future review of our work.

Sincerely,
Paolo Forni

Berke B, Wittnam J, McNeill E, Van Vactor DL, Keshishian H (2013) Retrograde BMP signaling at the synapse: a permissive signal for synapse maturation and activity-dependent plasticity. J Neurosci 33:17937-17950.

Chamero P, Katsoulidou V, Hendrix P, Bufe B, Roberts R, Matsunami H, Abramowitz J, Birnbaumer L, Zufall F, Leinders-Zufall T (2011) G protein G(alpha)o is essential for vomeronasal function and aggressive behavior in mice. Proc Natl Acad Sci U S A 108:12898-12903.

© 2020. Published by The Company of Biologists under the terms of the Creative Commons Attribution License (https://creativecommons.org/licenses/by/4.0/).

Colak D, Mori T, Brill MS, Pfeifer A, Falk S, Deng C, Monteiro R, Mummery C, Sommer L, Gotz M (2008) Adult neurogenesis requires Smad4-mediated bone morphogenic protein signaling in stem cells. J Neurosci 28:434-446.

da Silva S, Hasegawa H, Scott A, Zhou X, Wagner AK, Han BX, Wang F (2011) Proper formation of whisker barrelettes requires periphery-derived Smad4-dependent TGF-beta signaling. Proc Natl Acad Sci U S A 108:3395-3400.

Hodge LK, Klassen MP, Han BX, Yiu G, Hurrell J, Howell A, Rousseau G, Lemaigre F, Tessier-Lavigne M, Wang F (2007) Retrograde BMP signaling regulates trigeminal sensory neuron identities and the formation of precise face maps. Neuron 55:572-586.

Hong W, Mosca TJ, Luo L (2012) Teneurins instruct synaptic partner matching in an olfactory map. Nature 484:201-207.

Montani G, Tonelli S, Sanghez V, Ferrari PF, Palanza P, Zimmer A, Tirindelli R (2013) Aggressive behaviour and physiological responses to pheromones are strongly impaired in mice deficient for the olfactory G-protein -subunit G8. J Physiol 591:3949-3962.

Prince JE, Brignall AC, Cutforth T, Shen K, Cloutier JF (2013) Kirrel3 is required for the coalescence of vomeronasal sensory neuron axons into glomeruli and for male-male aggression. Development 140:2398-2408.

Retting KN, Song B, Yoon BS, Lyons KM (2009) BMP canonical Smad signaling through Smad1 and Smad5 is required for endochondral bone formation. Development 136:1093-1104.

Sudhof TC (2017) Synaptic Neurexin Complexes: A Molecular Code for the Logic of Neural Circuits. Cell 171:745-769.

Tong KK, Kwan KM (2013) Common partner Smad-independent canonical bone morphogenetic protein signaling in the specification process of the anterior rhombic lip during cerebellum development. Mol Cell Biol 33:1925-1937.

Wang X, Harris RE, Bayston LJ, Ashe HL (2008) Type IV collagens regulate BMP signalling in Drosophila. Nature 455:72-77.

<u>Rebuttal response letter</u>

MS ID#: DEVELOP/2019/184036

MS TITLE: Smad4 signaling establishes the somatosensory map of basal vomeronasal sensory neurons

AUTHORS: Ankana Naik, Jennifer M. Lin, Ed Zandro M. Taroc, Raghu Katreddi, Jesus A. Frias, Morgan A. Sammons, and Paolo E. Forni

I have now received two further referees' reports on the above manuscript, and have reached a decision on your Rebuttal request. The referees' comments are appended below, or you can access them online: please go to BenchPress and click on the 'Manuscripts with Decisions' queue in the Author Area.

As you will see, on balance all four of the referees express considerable interest in your work, but have some significant criticisms and recommend a substantial revision of your manuscript before we can consider publication. You will see that all of them recommend asubstantial re-writing of the manuscript to try and make your finding clearer. If you are able to revise the manuscript along the lines suggested, which may involve further experiments, I will be happy receive a revised version of the manuscript. Your revised paper will be re-reviewed by one or more of the original referees (I will exclude the most negative reviewer), and acceptance of your manuscript will depend on your

© 2020. Published by The Company of Biologists under the terms of the Creative Commons Attribution License (https://creativecommons.org/licenses/by/4.0/).

addressing satisfactorily the reviewers' major concerns. Please also note that Development will normally permit only one round of major revision.

Please attend to all of the reviewers' comments and ensure that you clearly highlight all changes made in the revised manuscript. Please avoid using 'Tracked changes' in Word files as these are lost in PDF conversion. I should be grateful if you would also provide a point-by-point response detailing how you have dealt with the points raised by the reviewers in the 'Response to Reviewers' box. If you do not agree with any of their criticisms or suggestions please explain clearly why this is so.

Reviewer 3:

I was kindly asked by the Editors of Development to assess if this manuscript would be suitable for a possible resubmission after it was rejected following the reviewing process. For this reason, I deliberately did not read the other reviewers' comments until completion of the analysis of the manuscript. In my personal opinion, this manuscript is undoubted interesting and reports solid information on the effect of BMP signaling deletion into two temporal phases of the VNO neurogenesis, thus offering a substantial contribution to the knowledge in this research field. Data are solid, well presented and follow a logical experimental strategy. The effects of BMP signaling deletions are convincing (and quantitatively in line with the results reported by other authors with the deletion of different signal transduction molecules). Images are very nice and instructive (except for figs. 2D1, 2E1, 2F1 that are not well defined), as much as the histological part that is accurately performed. However, this manuscript is weak on the interpretation of the results. For example, in the Discussion, the authors do not propose clear models on how BMP signaling may be working during the different phases of the neuronal maturation. Thus, a general reader is not assisted to recapitulate the effects of BMP signaling deletion with the mechanisms underlying VSN maturation. I do think that revisiting the Discussion and making it less technical will much improve the quality of the manuscript. I would also suggest introducing an illustrative cartoon as a final figure to make everything more understandable (see Fig 2SA). I would personally believe that this manuscript should have the chance to be considered for resubmission.

Reviewer 4:

After a close examination of the manuscript and review reports and, while not necessarily subscribing to each and every opinion expressed (particularly by reviewer #2), I found that they provide a fair overall assessment. Although I do not agree with reviewer #2's view about the manuscript weaknesses (too descriptive, modest changes, low significance to field), I do not believe there have been serious errors or misunderstandings on her/his part. The opinions expressed by the reviewer are subjective (so as mine), but the point is that the report contains no major mistakes and experiments were correctly interpreted. My opinion about the paper is that the subject and results are timely and interesting, but I have serious concerns about how results are presented, described and interpreted in the text, too many speculative claims and clarity of writing in general. In my view, the overall quality (and quantity) of the results is excellent. Authors also made an effort to use different approaches to validate most relevant results. I fundamentally agree with reviewer #1: presented data are novel, important to the field and need to be published. However, I also believe that the manuscript fails to focus on the importance/novelty of the findings, which for me are the role(s) of Smad4 in glomerular organization, VNE homeostasis and differential control of axon targeting on basal/apical VSNs. As suggested by reviewer #2, I think that Fig 1 is somehow misleading: expression data on other Smad genes and vasculature markers seem merely accessory and not straight to the point. The use of the term "gradient" is not very appropriate considering that only two epithelial layers exist. Similarly, "somatosensory" is out of context. In addition, the text contains several statements clearly overstating conclusions that are not fully supported by the data. For example, claims about "compromised functionality" of neurons based only on Fos expression; "neuronal survival" when not a single cell-death vs. proliferation study is present; or "presynaptic defects" in glomerular innervation using only indirect evidence appear disproportionate. In summary, the work is interesting, the data are solid and experiments are careful and thorough. However, some of the conclusions are not supported by the results. In my view, this paper should be strengthened by addressing a major review of these concerns as outlined

© 2020. Published by The Company of Biologists under the terms of the Creative Commons Attribution License (https://creativecommons.org/licenses/by/4.0/).

above, including a full revision of the title, abstract, results and discussion sections to be acceptable.

First revision

Author response to reviewers' comments

Reviewer 1

Suggestions for improving clarity:

1) Neither the abstract nor the introduction communicate the importance of the findings. They simply say SMAD4 is necessary, but what makes this novel and how does it address specificity? The reader needs to be exposed to the significance of the work early in the manuscript.

-We thank the reviewer for this comment. We extensively changed the abstract, introduction and discussion to better contextualize the rational and significance of the experimental outcomes. Please see tracked changes.

2) Localization of active BMP signaling section. Fig 1A&B: The RT-PCR and RNAseq levels of the ligand transcripts often do not match – this should be discussed.

-The RT-PCR presented in figure 1 are PCR from cDNA, not semi-quantitative PCRs as stated in S. TB1. We apologize for this miscommunication in supplementary table1. The meaning of the gel in figure 1 is to show that the amplicons we obtained were of the expected size as indicated in (S. TAB1). S.TAB1 has been corrected and the meaning of the figure has been better explained in the main text. (Specific Line numbers 90-93)

3) The rationale for the various analyses needs to be more precise. For example, analyzing the Arx-1 null mice is not
clearly explained; it may be obvious to a neuroscientist but perhaps not to a more generalized readership. Overall, the rationale for the observations reported in each "results" section needs to be clearer, and the conclusions should include the significance of these findings. The authors need to tell me what makes these findings important.

-We believe that this revised version better explains the rationale for experiments, procedures and significance of the outcomes. In particular Arx-1, the meaning of the observations on Arx-1 are now explained more clearly (Specific Line number 131-137, all changes are highlighted in the tracked version of the manuscript that we uploaded at supplementary documents.).

4) Same comment for the discussion section.

-Discussion has been extensively rewritten.

Minor points:
1) I am not sure that the DevBio community would understand that male urine contains pheromones – the rationale for this stimulus should be briefly explained.

-We have now provided additional information to also appeal to generalists.

2) "data" are plural – this needs to be fixed here and there in the manuscript.

-We corrected.

© 2020. Published by The Company of Biologists under the terms of the Creative Commons Attribution License (https://creativecommons.org/licenses/by/4.0/).

Reviewer 3

Main criticisms
1)Images are very nice and instructive (except for figs. 2D1, 2E1, 2F1 that are not well defined), as much as the histological part that is accurately performed.

-We thank the reviewer for appreciating our illustrations. We have now increased the contrast in Fig 2D1,2E1 and 2F1 to make the images more readable. Please note that the “low quality” of the fluorescence is because we performed the immunostaining after all the steps and developing the in-situ hybridization.

2)This manuscript is weak on the interpretation of the results. For example, in the Discussion, the authors do not propose clear models on how BMP signaling may be working during the different phases of the neuronal maturation. Thus, a general reader is not assisted to recapitulate the effects of BMP signaling deletion with the mechanisms underlying VSN maturation.

-We thank the reviewer for this criticism. We extensively rewrote the discussion giving more elements for data interpretation and relating more our results to the existing literature (Specific Line number 418-547).

3)I do think that revisiting the Discussion and making it less technical will much improve the quality of the manuscript. I would also suggest introducing an illustrative cartoon as a final figure to make everything more understandable (see Fig 2SA) discussion giving a better understanding of our data interpretation.

-We rewrote the discussion. We believe this revised version better explains the rationale and meaning of the experiments. We added a summary figure as panels J-K of figure 7.

Reviewer 4:

1)Fig 1 is somehow misleading: expression data on other Smad genes and vasculature markers seem merely accessory and not straight to the point. The use of the term “gradient” is not very appropriate considering that only two epithelial layers exist.

-In the revised version of the manuscript, we believe we better explained the meaning of figure1 in results. We believe that talking about signaling gradient referring to figure 1 is not inappropriate, as we observed and quantified different intensities of Smad activations (pSmad1,5,8) within the basal territories of the VNO (see lines 104-124). Moreover, we describe that the intensity of active BMP signal decreases proportionally to relative distance of the neurons with respect to the sources of collagen IV. We believe this is an important observation, as it shows that the neurons in the basal territories are experiencing different levels of morphogenic signals. We further discuss the significance of this result in the discussion.

2)Similarly, “somatosensory” is out of context.

-We removed the word “somatosensory” throughout the manuscript.

3)In addition, the text contains several statements clearly overstating conclusions that are not fully supported by the data. For example, claims about “compromised functionality” of neurons based only on Fos expression;

-We respectfully disagree, we tested pS6 activation in the primary epithelium and cFOS at postsynaptic level. pS6 is an accepted readout for VSNs activation in response to urine stimuli (Silvotti et al., 2018). The lack of cFOS activation in the AOB confirms the lack of signal transmission to the postsynaptic partners.

However, as the data that we presented did not seem to convince the reviewer we decided to dig a bit deeper and by doing that we found a new exciting phenotype. The new results are included in a paragraph (255-286) and in the figure 4 P-U, new section of material and methods and new Supplementary figures S3A,B.

© 2020. Published by The Company of Biologists under the terms of the Creative Commons Attribution License (https://creativecommons.org/licenses/by/4.0/).

In summary we found that AP-2εCreSmad4 mutants have dramatic defects in dendritic knobs. We believe these new data complete our previous observation and further increase the advance of our findings.

4) "neuronal survival" when not a single cell-death vs. proliferation study is present;

-As noted in the manuscript, we performed proliferation and cell death essays for all animals at all stages. Even though we did not find acute cell death, we did observe that 2 months after birth the AP-2εCreSmad4 mutants possess between 30 to 50% less basal neurons than controls (Fig 4 A-I). Using genetic lineage tracing, we demonstrated that these neurons do not transdifferentiate (Fig 4K-L1). Although we did not discover how they die, it is obvious that they do. For scientific rigor throughout the paper, we discuss the reduction in cell number over time rather that cell death. Please note that AP-2εis not expressed in proliferative progenitors, so the effects of Smad4 ablation are limited to postmitotic cells.
We now better clarified this point in Line (236-253) and in discussion (436-454)

5)… "presynaptic defects" in glomerular innervation using only indirect evidence appear disproportionate.

-With AP-2εcre and OMPcre manipulations, we removed Smad4 only in presynaptic basal VSNs. There is no recombination in the (postsynaptic) mitral cells in posterior AOB. Moreover, our data showing defective dendritic knobs of the basal VSNs support our statement that the functional defects arise from the cells in the epithelium.
In addition to this, in the new version of the manuscript we now provide new experimental data that support a loss-of-function in the VSNs in the epithelium (Specific Line numbers 255-286).

6)In my view, this paper should be strengthened by addressing a major review of these concerns as outlined above, including a full revision of the title, abstract, results and discussion sections to be acceptable.

-All points have been addressed, title, abstract and discussion have been extensively rewritten.

Silvotti, L., Cavaliere, R. M., Belletti, S. and Tirindelli, R. (2018). In-vivo activation of vomeronasal neurons shows adaptive responses to pheromonal stimuli. Sci Rep 8, 8490.

Third decision letter

MS ID#: DEVELOP/2019/184036

MS TITLE: Smad4 dependent morphogenic signals control basal vomeronasal sensory neurons' maturation and axonal targeting to the accessory olfactory bulb.

AUTHORS: Ankana S Naik, Jennifer M. Lin, Ed Zandro M. Taroc, Raghu Ram Katreddi, Jesus A. Frias, Alex A Lemus, Morgan A. Sammons, and Paolo E. Forni

I have now received all the referees reports on the above manuscript, and have reached a decision. The referees' comments are appended below, or you can access them online: please go to BenchPress and click on the 'Manuscripts with Decisions' queue in the Author Area.

The overall evaluation is positive and we would like to publish a revised manuscript in Development, provided that the referees' minor comments can be satisfactorily addressed. Please attend to all of the reviewers' comments in your revised manuscript and detail them in your point-by-point response. If you do not agree with any of their criticisms or suggestions explain clearly why this is so.

© 2020. Published by The Company of Biologists under the terms of the Creative Commons Attribution License (https://creativecommons.org/licenses/by/4.0/).

Reviewer 1

Advance summary and potential significance to field

Understanding how various signaling molecules regulate the specific outgrown and synaptic connections of axons to their targets is a major question in developmental neuroscience. One of the best studied systems in mammals is the olfactory sensory neuron connectivity to glomeruli targets in the olfactory bulb, which allows perception of odorants in space. Many animals, including humans, also have an accessory system for detecting pheromones, known as the vomeronasal organ (VNO). Although this system plays a key role in multiple behaviors it is much less studied. An RNAseq study found that multiple components of the TFG-beta family are highly expressed in the mouse postnatal VNO. To follow this clue, the authors used novel Cre-lines of mice to investigate whether TGF-beta signaling impacts the specificity of vomeronasal sensory neuron projections to the accessory olfactory bulb. This is an important question, the premise for the study is valid.

Comments for the author

The authors have made many improvements in the manuscript that now highlight the rationale for experimental approaches and significance of the findings. There are some textual inconsistencies that the proof editors will catch, but in addition a few corrections are needed:
1) Figure 1A is labeled as protein but the figure legend and text say these are transcript reads.
2) Line 314: explain the rationale for cFOS immunostaining
3) Line 348: explain the rationale for Ki67 immunostaining
4) Lines 392-393 sentence included a confusing double negative: none were not altered
5) Lines 409-416: define the known function of Kirrel family proteins
6) Introduction para1 (line 31): define VNO
7) Introduction para1 (line 37): define VSNs
8) Introduction para1 (line 39): VNE was already defined in line 33
9) Line 60, capitalize and italicize “drosophila”

Reviewer 3

Advance summary and potential significance to field

-

Comments for the author

After the substantial rewriting of some sections and the inclusion of more data, I do believe that this manuscript gives a remarkable contribution to the knowledge in this research field. I have few little suggestions to improve the overall quality of this paper:
1) Please check that the mouse genotype of “control” in the captions of figures containing histogram bars and in the Results (line 334, for example) is indicate, at least once.
2) Line 811, I guess villin should be lower case letters

Reviewer 4

Advance summary and potential significance to field

I have now re-reviewed the paper by Naik et al. The revised manuscript has been greatly improved. The authors have even included new set of data to address the criticisms.

Comments for the author

My remaining comments concerns the interpretation of some of the results:

© 2020. Published by The Company of Biologists under the terms of the Creative Commons Attribution License (https://creativecommons.org/licenses/by/4.0/).

1. Using immunostaining and a transgenic YFP mouse the authors convincingly show p-Smad1,5,8 labeling only basal VSNs. They also show a “negative correlation between the expression of p-Smad1/5/8 and the distance from the basement membrane” (Fig.1 K). This can be interpreted because cells more distant from the basal lamina are perhaps in a later stage of maturation. However, relative apical/basal cell position in the epithelium seems to also be determined by receptor-type expression according to Ishii and Mombaerts (J Neurosci 2008). Authors should discuss this possibility.

2. Several parts of the text still mention “cell loss”, “progressive cell death”, “decrease cell survival”, likely “related to impaired functionality”, but without a clear evidence of increase in apoptosis. A reduced proliferation rate or differentiation to basal VSNs could perhaps be an alternative explanation.

3. The authors claim that defective knob formation is responsible for the reduced response to male stimuli and causes the reduction of basal VSNs over time. I find this argument very speculative. First, TMEM16A signal amplification appears to be dispensable for the detection of male-specific pheromones (Münch et al, J Biol Chem 2018). Second, many reasons may account for the reduced sensitivity in dependent of knob morphology. For example, by reduced cell-type specific neurons, and/or differential expression of signaling molecules (including receptors) typical of mature neurons, such as Plcb2 and Gnai2, which have been shown to participate in male pheromone signaling and upregulated in the Smad4 KO. These alternatives should be discussed.

4. Similarly, the claim that reduction in cFOS activation in the aAOB reflects a post-synaptic/mitral cell defect is vaguely supported. No single evidence of mitral cell defect is shown.

Second revision

Author response to reviewers' comments

Point-by-point response
The line numbers mentioned in the responses are the ones of the “clean version” of the manuscript. In this version of the manuscript all the changes we made after to the reviewers’ suggestions have been highlighted in yellow.

Additional text edits can be seen in the tracked changes version of the manuscript that we uploaded as part of the supplementary files.

Figure 1 and Figure 4 had been replaced with corrected versions.

REVIEWER 1

The authors have made many improvements in the manuscript that now highlight the rationale for experimental approaches and significance of the findings. There are some textual inconsistencies that the proof editors will catch, but in addition a few corrections are needed:

1) Figure 1A is labeled as protein but the figure legend and text say these are transcript reads.
We changed the heading for Figure 1A.

2) Line 314: explain the rationale for cFOS immunostaining
We better explained the reationale, please see line numbers 305-306.

3) Line 348: explain the rationale for Ki67 immunostaining.
We believe that the reviewer either mistyped the line number or the antigen. Line 348 is about pS6. We already explained the rational of using pS6 as a marker in lines 237-240. We now also specified that Ki67 it is a staining for proliferating progenitors (lines 339-340).

© 2020. Published by The Company of Biologists under the terms of the Creative Commons Attribution License (https://creativecommons.org/licenses/by/4.0/).

4) Lines 392-393 sentence included a confusing double negative: none were not altered
We changed that particular sentence. Please see line number 382-383.

5) Lines 409-416: define the known function of Kirrel family proteins
We now specified that Kirrel proteins are adhesion molecules line number 399-400

6) Introduction para1 (line 31): define VNO
We already defined VNO in the abstract however for reader's convenience we also added definition in the introduction.

7) Introduction para1 (line 37): define VSNs
We already defined VSN in the abstract however for reader's convenience we also added definition in the introduction.

8) Introduction para1 (line 39): VNE was already defined in line 33
We changed it.

9) Line 60, capitalize and italicize "drosophila"
We changed it.

REVIEWER 3

After the substantial rewriting of some sections and the inclusion of more data, I do believe that this manuscript gives a remarkable contribution to the knowledge in this research field. I have few little suggestions to improve the overall quality of this paper:
1) Please check that the mouse genotype of "control" in the captions of figures containing histogram bars and at least once in the results (e.g.line 334).

The genotype for control and mutant mice is mentioned in results (e.g line numbers 208, 336), in Material and Methods (line number 551, 605) and in all figure legends (line numbers 754, 770, 783, 797, 817, 840). Figure 4 has multiple controls, therefore, we included the genotype of controls in Fig4 A,K,P and legend.

2) Line 811, I guess VILLIN should be lower case letters We changed.

REVIEWER 4

1. Using immunostaining and a transgenic YFP mouse the authors convincingly show p- Smad1,5,8 labeling only basal VSNs. They also show a "negative correlation between the expression of p-Smad1/5/8 and the distance from the basement membrane" (Fig.1 K). This can be interpreted because cells more distant from the basal lamina are perhaps in a later stage of maturation. However, relative apical/basal cell position in the epithelium seems to also be determined by receptor-type expression according to Ishii and Mombaerts (J Neurosci 2008). Authors should discuss this possibility.

From unpublished observations we made on published BrDU birth dating experiments <u>(Lin et al., 2018),</u> we never observed obvious relationship between cell body position in the distinct basal sublayers and the maturation stage/age of VSNs, therefore we do not think that the age related positioning of VSNs interpretation proposed by the reviewer should be included.
<u>However, as suggested by the reviewer we now quoted in discussion (line 507-510) the paper of Ishii and Mombaerts as this suggests that the cell bodies of VSNs expressing different receptors are distributed in different sublayers of the basal territory.</u>

2. Several parts of the text still mention "cell loss", "progressive cell death", "decrease cell survival", likely "related to impaired functionality", but without a clear evidence of increase in apoptosis. A reduced proliferation rate or differentiation to basal VSNs could perhaps be an alternative explanation.

© 2020. Published by The Company of Biologists under the terms of the Creative Commons Attribution License (https://creativecommons.org/licenses/by/4.0/).

-As noted in the manuscript, and in our previous rebuttal we did perform proliferation and cell death assays (Fig 3A-C). In all the mutants where the VSNs do not function properly, progressive cell loss has been reported (Chamero et al., 2011; Montani et al., 2013; Stowers et al., 2002). In our mutants the VSNs do not respond to stimuli as controls (Fig 4M-O).
Even though we did not find acute cell death, we observed that AP-2εCreSmad4 mutants had equal number of cells as in controls at 2 weeks after birth, however, at 2 months these mutants possessed between 30 to 50% less basal neurons than controls (Fig 4 A-I). So, the cells can form and differentiate normally but are progressively lost.
Please, note that AP-2εCre is not expressed in proliferative progenitors (Lin et al., 2018), so the effects of Smad4 ablation are limited to postmitotic cells. In addition to this when we quantified cell proliferation we found increased cell divisions in the mutants (Fig.3 C) this supports compensation to cell loss (Lin et al., 2018). We believe that these data rule out the alternative explanation proposed by the reviewer. Moreover, using genetic lineage tracing, we demonstrated that these neurons do not transdifferentiate (Fig 4K-L1). Although we did not discover how they die, it is obvious to us that they do.

3. The authors claim that defective knob formation is responsible for the reduced response to male stimuli and causes the reduction of basal VSNs over time. I find this argument very speculative. First, TMEM16A signal amplification appears to be dispensable for the detection of male-specific pheromones (Münch et al, J Biol Chem 2018).

In the paper by Münch et al, it is true that they showed no behavioral outcomes after TMEM16A loss, however, they showed functional defects of VSNs revealed by defective calcium currents in TMEM16A null VSNs. The fact that in AP-2εCreSmad4 mutants knobs are abnormal, TMEM16A is not expressed in the basal neurons, and neurons fail to get activated in response to stimuli, support a defective development and reduced functionality of these sensory neurons.

Second, many reasons may account for the reduced sensitivity independent of knob morphology. For example, by reduced cell-type specific neurons, and/or differential expression of signaling molecules (including receptors) typical of mature neurons, such as Plcb2 and Gnai2, which have been shown to participate in male pheromone signaling and upregulated in the Smad4 KO. These alternatives should be discussed.

Gnai2, is expressed by mature apical neurons not by mature basal neurons. In the AP-2e mutants we genetically manipulated the basal neurons only, not the apical neurons. In line with this we did not see defects in the apical population but reduction of the basal VSNs that are the neurons that have knob defects. The increase in Gnai2 mRNA could likely reflects a negative selection of basal neurons and an enrichment in transcripts form apical VSNs. The contribution proposed by the reviewer of upregulation of Plcb2 *to the described phenotype is completely hypothetical therefore we do not believe that should be included.*
However, based on the previous and this last comment we now changed lines 278-279 with a broader statement: "Overall, these data suggest that aberrant gene expression and defective neuronal functionality precedes and likely causes the reduction of basal VSNs over time."

4. Similarly, the claim that reduction in cFOS activation in the aAOB reflects a post-synaptic/mitral cell defect is vaguely supported. No single evidence of mitral cell defect is shown.

The mitral cells in the anterior AOB and the basal VSNs are the only cells of the vomeronasal system that express AP-2ε. We now rephrased that statement (Line 313-316) to stress that this is an interpretation not a definitive conclusion: "The reduction in cFOS expression in activated mitral cells in the anterior AOB could indicate a postsynaptic defect secondary to Smad4 ablation in the mitral cells of the aAOB. In fact, we previously reported that some mitral cells of the AOB are positive for AP-2ε lineage (Lin et al., 2018)."

Chamero, P., Katsoulidou, V., Hendrix, P., Bufe, B., Roberts, R., Matsunami, H., Abramowitz, J., Birnbaumer, L., Zufall, F., and Leinders-Zufall, T. (2011). G protein G(alpha)o is essential for vomeronasal function and aggressive behavior in mice. Proc Natl Acad Sci USA *108*, 12898- 12903.

© 2020. Published by The Company of Biologists under the terms of the Creative Commons Attribution License (https://creativecommons.org/licenses/by/4.0/).

Lin, J.M., Taroc, E.Z.M., Frias, J.A., Prasad, A., Catizone, A.N., Sammons, M.A., and Forni, P.E. (2018). The transcription factor Tfap2e/AP-2epsilon plays a pivotal role in maintaining the identity of basal vomeronasal sensory neurons. Dev Biol *441*, 67-82.
Montani, G., Tonelli, S., Sanghez, V., Ferrari, P.F., Palanza, P., Zimmer, A., and Tirindelli, R. (2013). Aggressive behaviour and physiological responses to pheromones are strongly impaired in mice deficient for the olfactory G-protein -subunit G8. J Physiol *591*, 3949-3962.
Stowers, L., Holy, T.E., Meister, M., Dulac, C., and Koentges, G. (2002). Loss of sex discrimination and male-male aggression in mice deficient for TRP2. Science *295*, 1493-1500.

<u>Fourth decision letter</u>

MS ID#: DEVELOP/2019/184036

MS TITLE: Smad4 dependent morphogenic signals control basal vomeronasal sensory neurons' maturation and axonal targeting to the accessory olfactory bulb.

AUTHORS: Ankana S Naik, Jennifer M. Lin, Ed Zandro M. Taroc, Raghu Ram Katreddi, Jesus A. Frias, Alex A Lemus, Morgan A. Sammons, and Paolo E. Forni
ARTICLE TYPE: Research Article

I am happy to tell you that your manuscript has been accepted for publication in Development, pending our standard ethics checks.

© 2020. Published by The Company of Biologists under the terms of the Creative Commons Attribution License (https://creativecommons.org/licenses/by/4.0/).
